# Glutaminase-1 inhibition alleviates senescence of Wharton’s jelly-derived mesenchymal stem cells via senolysis

**DOI:** 10.1093/stcltm/szae053

**Published:** 2024-08-09

**Authors:** Eun Joo Lee, Sun Jeong Kim, Su Yeon Jeon, Soobeen Chung, Sang Eon Park, Jae‑Sung Kim, Suk-Joo Choi, Soo-Young Oh, Gyu Ha Ryu, Hong Bae Jeon, Jong Wook Chang

**Affiliations:** Cell and Gene Therapy Institute, ENCell Co. Ltd., Seoul 06072, Republic of Korea; Cell and Gene Therapy Institute, Samsung Medical Center, Seoul 06351, Republic of Korea; Cell and Gene Therapy Institute, ENCell Co. Ltd., Seoul 06072, Republic of Korea; Cell and Gene Therapy Institute, Samsung Medical Center, Seoul 06351, Republic of Korea; Cell and Gene Therapy Institute, ENCell Co. Ltd., Seoul 06072, Republic of Korea; Cell and Gene Therapy Institute, Samsung Medical Center, Seoul 06351, Republic of Korea; Cell and Gene Therapy Institute, ENCell Co. Ltd., Seoul 06072, Republic of Korea; Cell and Gene Therapy Institute, Samsung Medical Center, Seoul 06351, Republic of Korea; Cell and Gene Therapy Institute, ENCell Co. Ltd., Seoul 06072, Republic of Korea; Cell and Gene Therapy Institute, Samsung Medical Center, Seoul 06351, Republic of Korea; Division of Radiation Biomedical Research, Korea Institute of Radiological and Medical Sciences, Seoul 139706, Republic of Korea; Radiological and Medico‑Oncological Sciences, University of Science and Technology, Daejeon 34113, Republic of Korea; Department of Obstetrics and Gynecology, Samsung Medical Center, Seoul 06351, Republic of Korea; Department of Obstetrics and Gynecology, Samsung Medical Center, Seoul 06351, Republic of Korea; Department of Medical Device Management and Research, SAIHST, Sungkyunkwan University, Seoul 06355, Republic of Korea; The Office of R&D Strategy & Planning, Samsung Medical Center, Seoul 06351, Republic of Korea; Cell and Gene Therapy Institute, ENCell Co. Ltd., Seoul 06072, Republic of Korea; Cell and Gene Therapy Institute, ENCell Co. Ltd., Seoul 06072, Republic of Korea; Cell and Gene Therapy Institute, Samsung Medical Center, Seoul 06351, Republic of Korea; Department of Health Sciences and Technology, SAIHST, Sungkyunkwan University, Seoul 06355, Republic of Korea

**Keywords:** BPTES, GLS1, replicative senescence, senolysis, mesenchymal stem cells, Wharton’s jelly

## Abstract

Replicative senescence of mesenchymal stem cells (MSCs) caused by repeated cell culture undermines their potential as a cell therapy because of the reduction in their proliferation and therapeutic potential. Glutaminase-1 (GLS1) is reported to be involved in the survival of senescent cells, and inhibition of GLS1 alleviates age-related dysfunction via senescent cell removal. In the present study, we attempted to elucidate the association between MSC senescence and GLS1. We conducted in vitro and in vivo experiments to analyze the effect of GLS1 inhibition on senolysis and the therapeutic effects of MSCs. Inhibition of GLS1 in Wharton’s jelly-derived MSCs (WJ-MSCs) reduced the expression of aging-related markers, such as p16, p21, and senescence-associated secretory phenotype genes, by senolysis. Replicative senescence-alleviated WJ-MSCs, which recovered after short-term treatment with bis-2-(5-phenylacetamido-1,2,4-thiadiazol-2-yl)ethyl sulfide 3 (BPTES), showed increased proliferation and therapeutic effects compared to those observed with senescent WJ-MSCs. Moreover, compared to senescent WJ-MSCs, replicative senescence-alleviated WJ-MSCs inhibited apoptosis in serum-starved C2C12 cells, enhanced muscle formation, and hindered apoptosis and fibrosis in mdx mice. These results imply that GLS1 inhibition can ameliorate the therapeutic effects of senescent WJ-MSCs in patients with muscle diseases such as Duchenne muscular dystrophy. In conclusion, GLS1 is a key factor in modulating the senescence mechanism of MSCs, and regulation of GLS1 may enhance the therapeutic effects of senescent MSCs, thereby increasing the success rate of clinical trials involving MSCs.

## Graphical Abstract

**Graphical Abstract:**
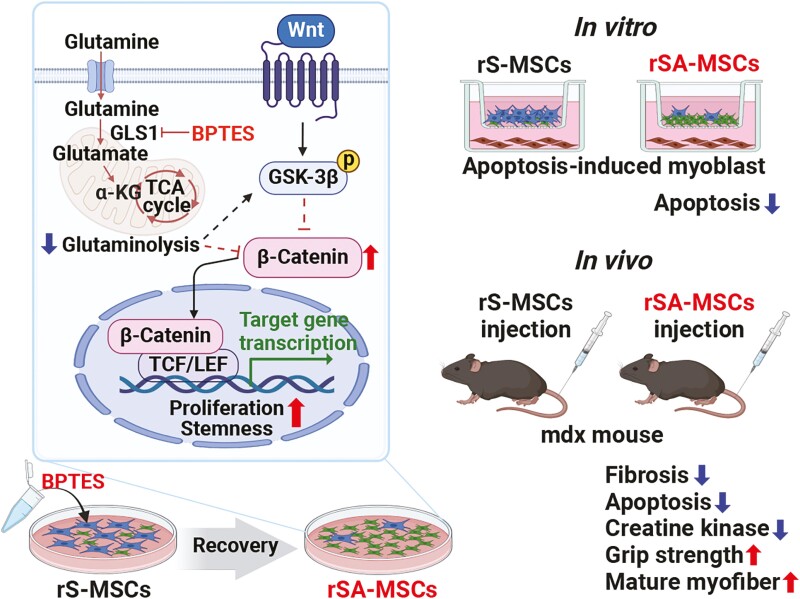

**Significance Statement**

This is the first study to demonstrate that glutaminase-1 (GLS1) could be a target for modulating replicative senescence in mesenchymal stem cells (MSCs). Through the regulation of GLS1, the proliferation and therapeutic efficiency of replicatively senescent MSCs can be enhanced. Therefore, this study is the first step toward developing technologies to control the replicative senescence observed during the mass production of MSCs.

## Introduction

Mesenchymal stem cells (MSCs) are used to treat various diseases owing to their self-renewal ability, multiple differentiation potential,^1^ and low immunogenicity.^2^ In previous studies, we demonstrated the therapeutic effects of MSCs by showing that MSCs alleviate symptoms in in vitro and in vivo models of various muscle diseases.^3-5^ However, to apply MSCs clinically, a sufficient number of cells must be obtained, and the cells must be cultured for a long period of time, which inevitably induces replicative senescence.^6^ MSC senescence is detrimental to the safety of MSC therapy and reduces therapeutic efficacy because of increased immunogenicity^7^ and reduced proliferation,^8^ migration,^9^ differentiation,^10^ and regeneration.^11^ Therefore, understanding the mechanism underlying MSC senescence is vital for the development of strategies for suppressing senescence and the effective application of MSCs in clinical trials.

Cellular senescence can be triggered by DNA damage,^12^ oxidative stress,^13^ chemotherapy,^14^ and mitochondrial dysfunction.^15^ As cellular senescence progresses, cells become large and flattened, and the lysosomal content increases.^16^ Replicative senescence leads to the cessation of proliferation^17^ and shortening of telomeres due to continuous cell division.^18^ In senescent cells, senescence-associated beta-galactosidase (SA-β-gal) activity increases after activation of p53/p21 and p16 pathways,^19^ and the expression of senescence-associated secretory phenotype (SASP) genes is enhanced, leading to accelerated senescence.^20^

Glutaminase-1 (GLS1) converts glutamine into glutamate during glutaminolysis.^21^ Recent studies have reported that GLS1 is concerned with the survival of senescent cells and that diverse age-related disease symptoms can be alleviated via glutaminolysis inhibition.^22,23^ In senescent cells, lysosomal permeability increases under conditions of lysosomal dysfunction, which leads to the loss of lysosomal acidity, resulting in cytosolic acidification.^24,25^ In fibroblasts, senescent cells enhance glutaminolysis by upregulating GLS1. Glutaminolysis neutralizes cytosolic acidity by promoting ammonia production, which is involved in senescent cell survival. Conversely, inhibition of GLS1 reduces age-related dysfunction by eliminating senescent cells^22^; GLS1 activity can be hindered by various inhibitors.^26^

Several strategies have been developed to suppress cellular senescence, among which senolytics specifically remove senescent cells. Chemical senolytic agents can inhibit senescence induction by inhibiting SASP secretion in senescent cells.^27^ However, the disadvantage of chemical senolytic agents is that the safety of the MSCs used for therapy may be compromised if the cells are cultured for a long time.^18^

In this study, we attempted to elucidate the association between MSC senescence and GLS1 expression, which has not been previously investigated. In addition, we confirmed that GLS1 inhibitors improved the therapeutic efficacy of senescent MSCs via senolysis, both in vitro and in vivo.

## Materials and methods

### MSC culture

This study was conducted in accordance with the tenets of the World Medical Association’s Declaration of Helsinki. Umbilical cords were collected from pregnant women with prior consent, in accordance with the guidelines approved by the Institutional Review Board of Samsung Medical Center (IRB approval number: 2016-07-102-043). WJ-MSCs were separated as described previously.^3^ Bone marrow-derived MSCs (BM-MSCs) were purchased from Cambrex (Lonza, #PT-2501, Durham, NC, USA). MSCs were cultured in minimum essential medium alpha (Gibco, Carlsbad, CA, USA) supplemented with 10% fetal bovine serum (FBS; Gibco) and 0.5% gentamicin solution. Bis-2-(5-Phenylacetamido-1,2,4-thiadiazol-2-yl)ethyl sulfide 3 (BPTES; Sigma, St. Louis, MO, USA), compound 968 (C968; Sigma) and CB-839 (Selleckchem, Houston, TX, USA) were used as GLS1 inhibitors at concentrations of 30, 10, and 1 µM, respectively.

Mouse myoblast C2C12 cells (ATCC CRL-1772; American Type Culture Collection, Rockville, MD, USA) were seeded at a density of 10 000 cells/cm^2^ in 6-well plates, and WJ-MSCs were seeded at a density of 10 000 cells/cm^2^ in Transwell inserts (pore size, 1 µm; BD Biosciences, Franklin Lakes, NJ, USA). C2C12 cells were cultured with or without WJ-MSCs for 24 hours under serum starvation conditions.

### SA-β-gal staining

To confirm cellular senescence, we performed SA-β-gal staining using an SA-β-gal staining kit (Cell Signaling Technology, Beverly, MA, USA). Cells were seeded in 6-well plates in triplicates, washed with phosphate-buffered saline (PBS), and fixed at room temperature (RT) for 5 minutes. Subsequently, the cells were incubated at 37 °C with SA-β-gal staining solution (pH 6) for 18 hours. Images were acquired using a microscope (Olympus CKX41; Olympus, Tokyo, Japan), and β-gal-positive cells were quantified after normalization against total cell numbers using ImageJ (National Institutes of Health, Bethesda, MD, USA).

### Cell counting kit-8 assay

We seeded 1000 cells in 96-well plates and conducted a cell counting kit-8 (CCK-8) assay. These cells were incubated with 10 µL CCK-8 reagent (Dojindo, Tokyo, Japan) for 3 hours at 37 °C under 5% CO_2_. After 3 hours, the absorbance was read at 450 nm using a microplate reader (xMark Microplate Absorbance Spectrophotometer; Bio-Rad Laboratories, Inc, Hercules, CA, USA).

### Cell counting

Cells were seeded at a density of 2000 cells/cm^2^ in 6-well plates and incubated at 37 °C under 5% CO_2_. The cells were detached using 0.25% trypsin-EDTA (Gibco), diluted with trypan blue at a 1:1 ratio, and counted using a hemocytometer.

### Immunocytochemistry

The cells were fixed in 4% paraformaldehyde at RT for 5 minutes and permeabilized in PBS containing 0.25% Triton X-100 at RT for 5 minutes. Next, the cells were incubated for 1 hour at RT in blocking solution (2% bovine serum albumin and 0.1% Tween 20 prepared in PBS), and thereafter, incubated with primary antibodies overnight at 4 °C. The primary antibodies used were anti-Lamin B1 (Abcam, Cambridge, MA, USA) and anti-annexin V (Abcam). The cells were then incubated with secondary antibodies for 1 hour at RT and counterstained with 4ʹ,6-diamidino-2-phenylindole (DAPI; Invitrogen, Waltham, MA, USA). Images were captured using a fluorescence microscope (Olympus DP74; Olympus, Tokyo, Japan) and a confocal microscope (Zeiss LSM800; Carl Zeiss Meditec, Jena, Germany). Fluorescence intensity was quantified using ImageJ and normalized using DAPI staining.

### Small interfering RNA transfection

All small interfering RNAs (siRNAs) were purchased from Bioneer Corporation (Daejeon, Korea). The sequences of the GLS1-specific siRNAs are listed in Supplementary Table S1. WJ-MSCs were transfected with siRNAs using Lipofectamine RNAiMAX (Invitrogen), according to the manufacturer’s protocol. Transfection was performed by adding serum-free medium to the cell suspensions at a final concentration of 25 nM. The control was non-silencing scrambled RNA (siNC) with at least 4 mismatches with any human, rat, or mouse gene.

### Quantitative RT-PCR analysis

Total RNA was extracted using the AccuPrep Universal RNA Extraction Kit (Bioneer), and cDNA was synthesized using SuperScript IV (Invitrogen) according to the manufacturer’s instructions. The primer sequences are listed in Supplementary Table S2. Quantitative reverse transcription polymerase chain reaction (qRT-PCR) was performed with 2× Power SYBR Green Master Mix (Applied Biosystems, Thermo Fisher Scientific) on a QuantStudio 6 Flex platform (Thermo Fisher Scientific, Inc, Waltham, MA, USA). The expression levels of target genes were normalized to those of *GAPDH* in each sample using the 2^−ΔΔCt^ method.

### Western blot analysis

Total protein was extracted using radioimmunoprecipitation assay (RIPA) buffer (Biosesang, Sungnam, Korea) supplemented with protease inhibitor cocktail (GenDEPOT, Katy, TX, USA) and ethylenediaminetetraacetic acid (EDTA; GenDEPOT). Proteins were separated using 4%-12% sodium dodecyl sulfate-polyacrylamide gel electrophoresis (SDS-PAGE; Bio-Rad) and transferred onto a polyvinylidene fluoride (PVDF) membrane (Bio-Rad). The membranes were blocked for 1 hour at RT using 5% skim milk and incubated overnight at 4 °C with the primary antibodies; anti-beta-galactosidase-1 (anti-GLB1), anti-GLS1, and anti-fibronectin (Abcam), anti-p16, anti-p21, anti-cleaved poly-(ADP-ribose) polymerase (PARP), anti-cleaved Caspase 3, anti-annexin V, anti-β-catenin, anti-microtubule-associated protein 1 light chain 3 (LC3)B, anti-p-glycogen synthase kinase 3β (GSK3β; Cell Signaling), anti-GSK3β (Cell Signaling), anti-myosin heavy-chain (MHC; R&D, NE Minneapolis, USA), anti-α-tubulin (Sigma, St. Louis, MO, USA), anti-glyceraldehyde 3-phosphate dehydrogenase (GAPDH), and anti-β-actin (Santa Cruz Biotechnology, Dallas, TX, USA). Subsequently, the membranes were incubated with goat anti-mouse and anti-rabbit horseradish peroxidase (HRP)-conjugated secondary antibodies (Abclon, Seoul, Korea) for 1 hour at RT. The bands were detected using a gel imaging system (Amersham Imager 600; GE Healthcare, Buckinghamshire, UK). The expression levels of target proteins were normalized to those of housekeeping protein in each sample using ImageJ.

### Metabolite analysis

WJ-MSCs were grown in serum-free culture medium with DMSO or BPTES for 24 hours. Subsequently, the culture medium was used to measure the levels of glutamine with glutamine assay kits (Abnova, Taipei, Taiwan). Cell lysates were used to determine the amounts of glutamine, glutamate, and α-ketoglutarate using glutamine assay kits, glutamate assay kits, and α-ketoglutarate assay kit (Dojindo), respectively, according to the manufacturer’s instructions. Absorbance for glutamine and glutamate levels was measured using a microplate reader (xMark Microplate Absorbance Spectrophotometer). The fluorescence for α-ketoglutarate levels was measured using a Varioskan LUX multimode microplate reader (Thermo Fisher Scientific).

### Flow cytometry

WJ-MSCs were stained with the surface markers CD44, CD73, CD90, CD105, CD14, CD11b, HLA-DR (MHC-II), CD34, CD45, and CD19 (BD Biosciences) in fluorescence-activated cell sorting (FACS) buffer (2% FBS in Dulbecco’s PBS) for 30 minutes at RT in the dark. At least 10 000 events were recorded using a BD FACSVerse (BD Biosciences), and the results were analyzed using the BD FACSuite software (version 10; BD Biosciences).

### Animals

Wild-type (C57BL/10ScSnJ [JAX: 000476]) and C57BL/10ScSn-Dmd^mdx^/J (mdx; JAX: 001801) mice were purchased from The Jackson Laboratory (Bar Harbor, ME, USA); wild-type mice were used as controls. Mdx mice (4-5 months old) were injected with 5 × 10^4^ WJ-MSCs suspended in 100 µL PBS via the tail vein. Seven days after the injection, the mice were sacrificed using isoprene to harvest the muscle tissues and blood. Seven mice per group were used in each experiment. Injected cell dose and duration were determined based on our previous study.^4^

### Grip strength measurements

Grip strengths of the forelimb and hindlimb were measured using a grip strength meter (BIO GS3; Bioseb, Vitrolles, France) before and after MSC-injection. Grip strength was normalized according to body weight, and the preinjection strengths and postinjection strengths were compared.

### Creatine kinase assays

Serum was extracted from blood samples collected from the retro-orbital plexus, and the creatine kinase (CK) activity in the serum was determined using CPK-PIII (Fujifilm, Tokyo, Japan) and measured using a DRY CHEM 7000i analyzer (Fujifilm). The CK activity before and after MSC-injection was compared.

### Immunohistochemistry

Gastrocnemius muscles tissues were fixed with 4% paraformaldehyde, embedded in paraffin blocks, and sectioned into 4-µm-thick sections. The sections were stained with primary antibodies, namely, anti-MHC (R&D), anti-annexin V (Abcam), and anti-collagen-1 (Sigma), and then incubated with the secondary antibodies, Alexa Fluor 488 goat anti-mouse immunoglobulin G1 (IgG1), and Alexa Fluor 594 goat anti-rabbit IgG (H + L; Invitrogen). Images were taken using a fluorescence microscope (Axio Observer D1; Carl Zeiss Meditec). Relative intensity was normalized to that of DAPI. Sirius Red staining was conducted using a Picro-Sirius Red Staining Kit (Abcam), following the manufacturer’s instructions. Images were acquired using a ScanScope AT (Leica Biosystems, Deer Park, IL, USA).

### Transcriptome analysis

RNA was prepared using the AccuPrep Universal RNA Extraction Kit (Bioneer), and libraries were constructed using the QuantSeq 3ʹ mRNA-Seq Library Prep Kit (E-Biogen, Inc, Seoul, Korea). High-throughput sequencing was performed as single-end 75 sequencing using NextSeq 500 (Illumina, Inc., USA). Heatmaps were drawn using the MeV (Multiple Experiment Viewer) program (version 4.9.0; https://webmev.tm4.org/). Gene Ontology (GO) and Kyoto Encyclopedia of Genes and Genomes (KEGG) pathway analyses were conducted using DAVID (version 6.8). The top 10 GO terms and KEGG pathways were identified based on p-values (*P* < .05). Gene set enrichment analysis (GSEA) was performed using GSEA software (version 4.3.2.) based on GO (c5.go.bp. database). The significant gene sets of GESA use a nominal *P*-value ≤ .05 and a false discovery rate (FDR) ≤ 0.25.

### Statistical analysis

All statistical analyses were conducted in IBM SPSS Statistics 23 (IBM Corp, Armonk, NY, USA). A 2-tailed Student’s *t* test was used to compare the results of 2 groups, and one-way Analysis of Variance with Tukey’s or Dunnett’s multiple-comparison post hoc test was used to compare the results of more than 2 groups. Data are expressed as the mean ± SD. Statistical significance was set at a *P*-value < .05 (*), .01 (**), or .001 (***).

## Results

### Inhibition of GLS1 induces senolysis in WJ-MSCs

**Figure 1.**
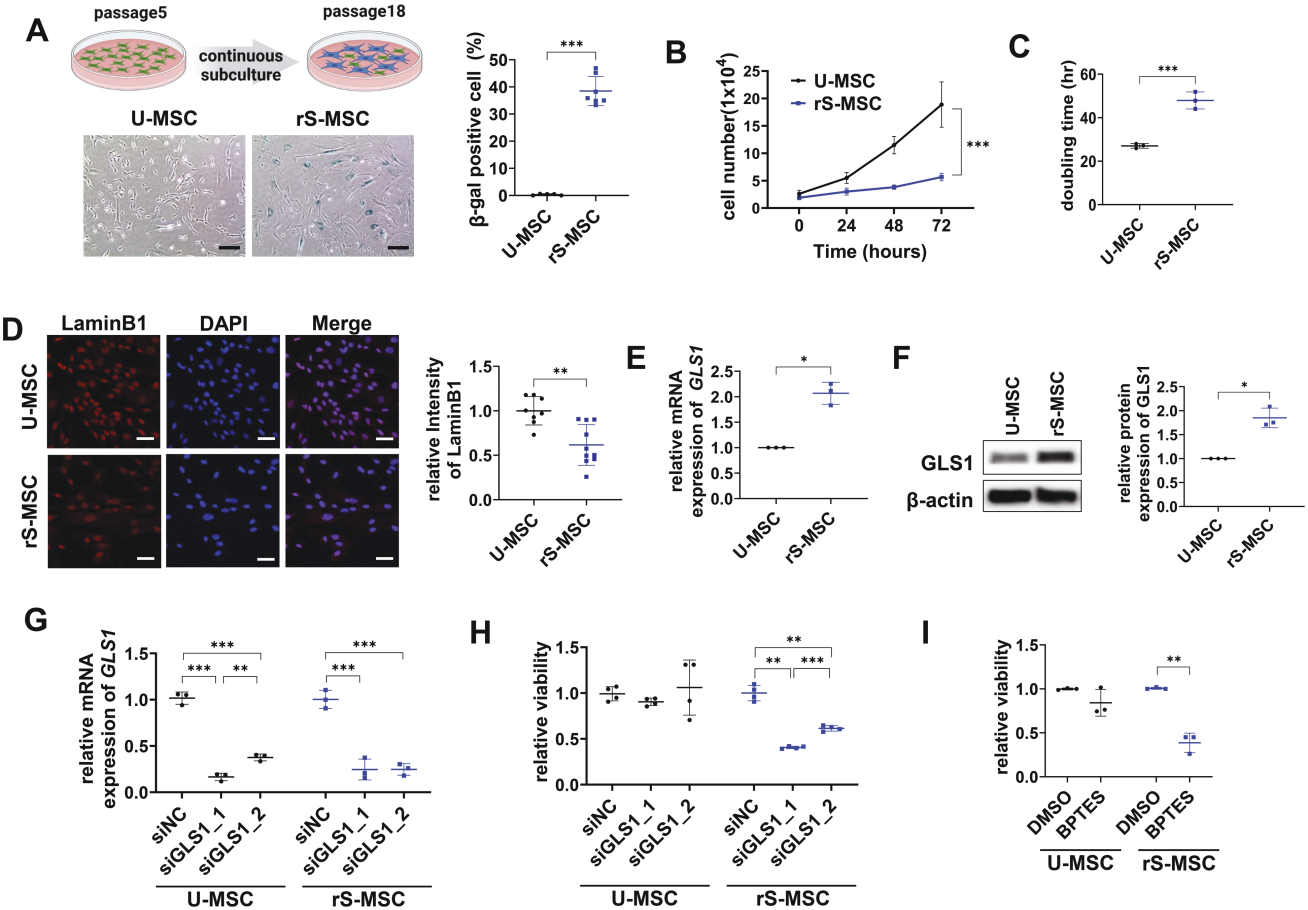
Reduced cell survival after glutaminase-1 (GLS1) inhibition in replicatively senescent Wharton’s jelly-derived mesenchymal stem cells (WJ-MSCs). (A) Senescence-uninduced MSCs (U-MSCs) and replicatively senescent MSCs (rS-MSCs) were stained with senescence-associated (SA)-β-galactosidase. Scale bar = 200 µm. (B) Cell numbers were counted 0, 24, 48, and 72 hours after cell seeding. (C) Doubling time was calculated by counting the numbers of U-MSCs and rS-MSCs. (D) Immunocytochemical analysis of laminB1 in U-MSCs and rS-MSCs. Scale bar = 50 µm. The chart was used to quantify the intensity of lamin B1 divided by the intensity of 4ʹ,6-diamidino-2-phenylindole (DAPI) staining. (E) qRT-PCR and (F) immunoblotting of U-MSCs and rS-MSCs for the confirmation of GLS1 expression. (G) Suppression of *GLS1* was examined by qRT-PCR after small interfering RNA (siRNA) transfection of U-MSCs and rS-MSCs. (H) Cell viability was examined 72 hours after siGLS1 transfection of U-MSCs and rS-MSCs. (I) Cell viability was measured after treating U-MSCs and rS-MSCs with dimethyl sulfoxide (DMSO) or bis-2-(5-phenylacetamido-1,2,4-thiadiazol-2-yl)ethyl sulfide 3 (BPTES; 30 µM) for 72 hours. Unpaired 2-tailed Student’s *t* test (A-F, I), one-way analysis of variance (ANOVA) with Tukey’s multiple comparisons post hoc test (G), and one-way ANOVA with Dunnett’s multiple comparisons post hoc test (H) were performed. ***P < .001, **P < .01, and *P < .05.

To induce replicative senescence, WJ-MSCs were continuously subcultured from passages 5 to 18. The proportion of SA-β-gal-positive cells among WJ-MSCs at passage 18 was significantly higher than that in WJ-MSCs at passage 5. Therefore, passage 5 WJ-MSCs were used as senescence-uninduced MSCs (U-MSCs), and passage 18 WJ-MSCs were used as replicatively senescent MSCs (rS-MSCs; Figure 1A). Compared to U-MSCs, rS-MSCs revealed reduced proliferation (Figure 1B, 1C) and decreased intensity of lamin B1, a structural protein of the nuclear lamina whose expression decreases in senescent cells (Figure 1D).^28^ These results confirmed the establishment of a replicative senescence model using WJ-MSCs.

In WJ-MSCs, GLS1 was remarkably upregulated in rS-MSCs compared to U-MSCs (Figure 1E, 1F). Therefore, senolysis was induced by the suppression of GLS1, which is involved in the survival of senescent cells. GLS1 knockdown and treatment with BPTES, a GLS1 inhibitor, effectively induced senolysis in WJ-MSCs (Figure 1G–1I).

### Inhibition of GLS1 reduces replicative senescence through senolysis

**Figure 2.**
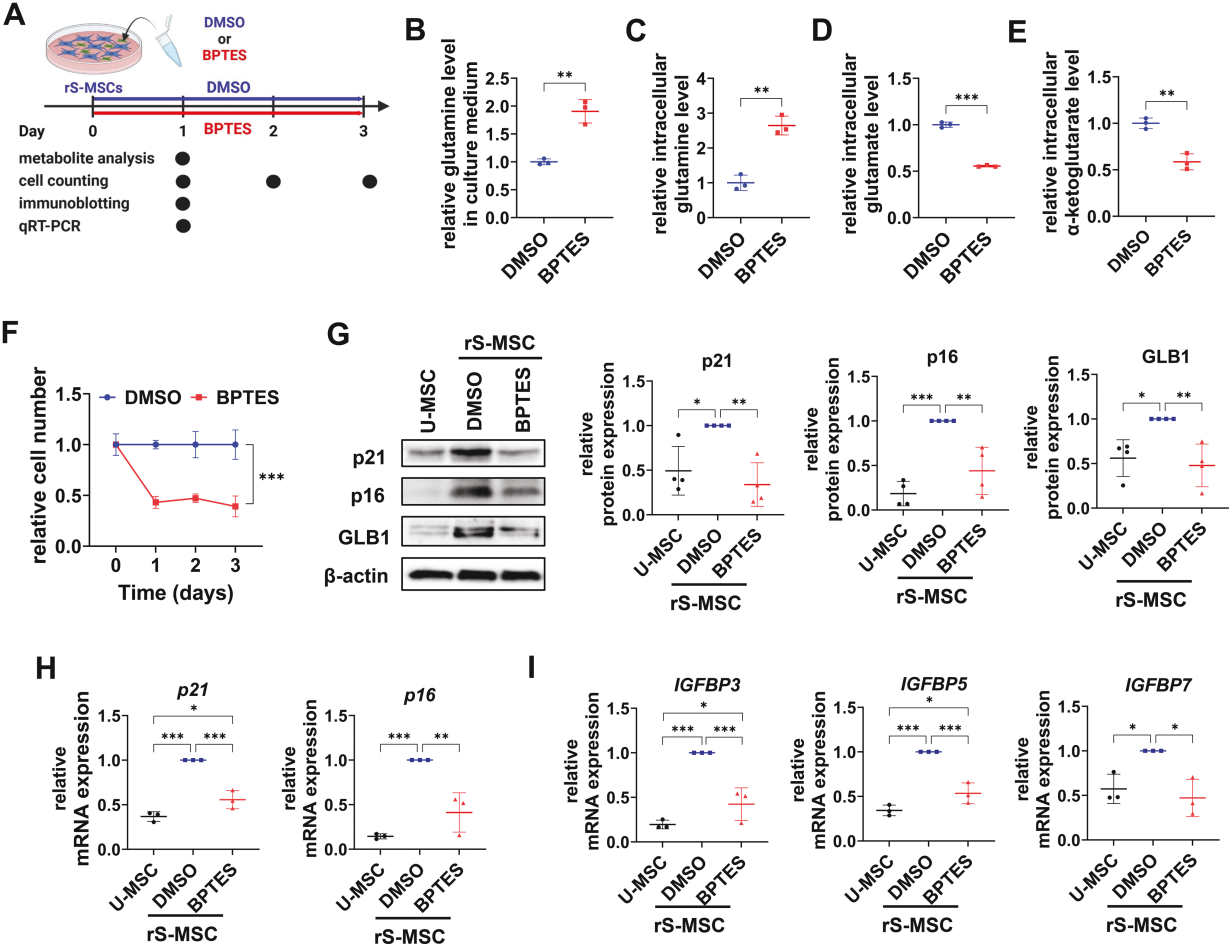
BPTES inhibits senescence of Wharton’s jelly-derived mesenchymal stem cells (WJ-MSCs). (A) Illustration of the experimental procedure. (B) Relative glutamine levels in the culture medium, (C) relative intracellular glutamine levels, (D) relative intracellular glutamate levels, and (E) relative intracellular α-ketoglutarate levels in which rS-MSCs were treated with DMSO or BPTES (30 µM) for 24 hours. (F) Relative cell numbers of the rS-MSCs treated with DMSO or BPTES (30 µM). (G) Levels of p21, p16, and beta-galactosidase-1 (GLB1) proteins in U-MSCs, rS-MSCs, and BPTES-treated rS-MSCs. (H) mRNA expression of *p21* and *p16* and (I) senescence-associated secretory phenotype (SASP) genes (insulin-like growth factor binding protein 3 [*IGFBP3*], *IGFBP5*, and *IGFBP7*) in U-MSCs, rS-MSCs, and BPTES-treated rS-MSCs. Two-tailed Student’s *t* test (B-F) and one-way ANOVA with Tukey’s multiple comparisons post hoc test (G-I) were performed. ***P < .001, **P < .01, and *P < .05.

Replicatively senescent MSCs were treated with BPTES to confirm the association between GLS1 and senescence in WJ-MSCs (Figure 2A). After BPTES treatment, glutamine, glutamate, and α-ketoglutarate levels were measured to confirm the inhibition of GLS1 activity. After BPTES treatment, glutamine levels increased both in the culture medium and in the cells (Figure 2B, 2C), and glutamate and α-ketoglutarate levels decreased in the cells (Figure 2D, 2E). This indicates that BPTES treatment was appropriate for the inhibition of glutaminolysis. Additionally, BPTES drastically decreased the viability of rS-MSCs after 24 hours (Figure 2F).

The levels of p16, p21, and GLB1, which were increased due to senescence, were significantly inhibited upon BPTES treatment (Figure 2G, 2H). In rS-MSCs, the mRNA levels of SASP genes, such as *insulin-like growth factor binding protein 3* (*IGFBP3*), *IGFBP5*, and *IGFBP7*, were also reduced after BPTES treatment (Figure 2I). Small interfering RNA transfection revealed that GLS1 plays an important role in inhibiting replicative senescence in WJ-MSCs (Supplementary Figure S1). Other GLS1 inhibitors, such as CB-839 and C968, effectively reduced the expression of senescence-related genes (Supplementary Figure S2). In addition, the stemness, which was reduced due to senescence, increased after BPTES treatment (Supplementary Figure S3). The results indicate that GLS1 is a key factor controlling senescence in WJ-MSCs, and that its inhibition caused senolysis.

### The anti-apoptotic effects on serum-starved C2C12 cells are increased in replicative senescence-alleviated MSCs compared to replicatively senescent MSCs

**Figure 3.**
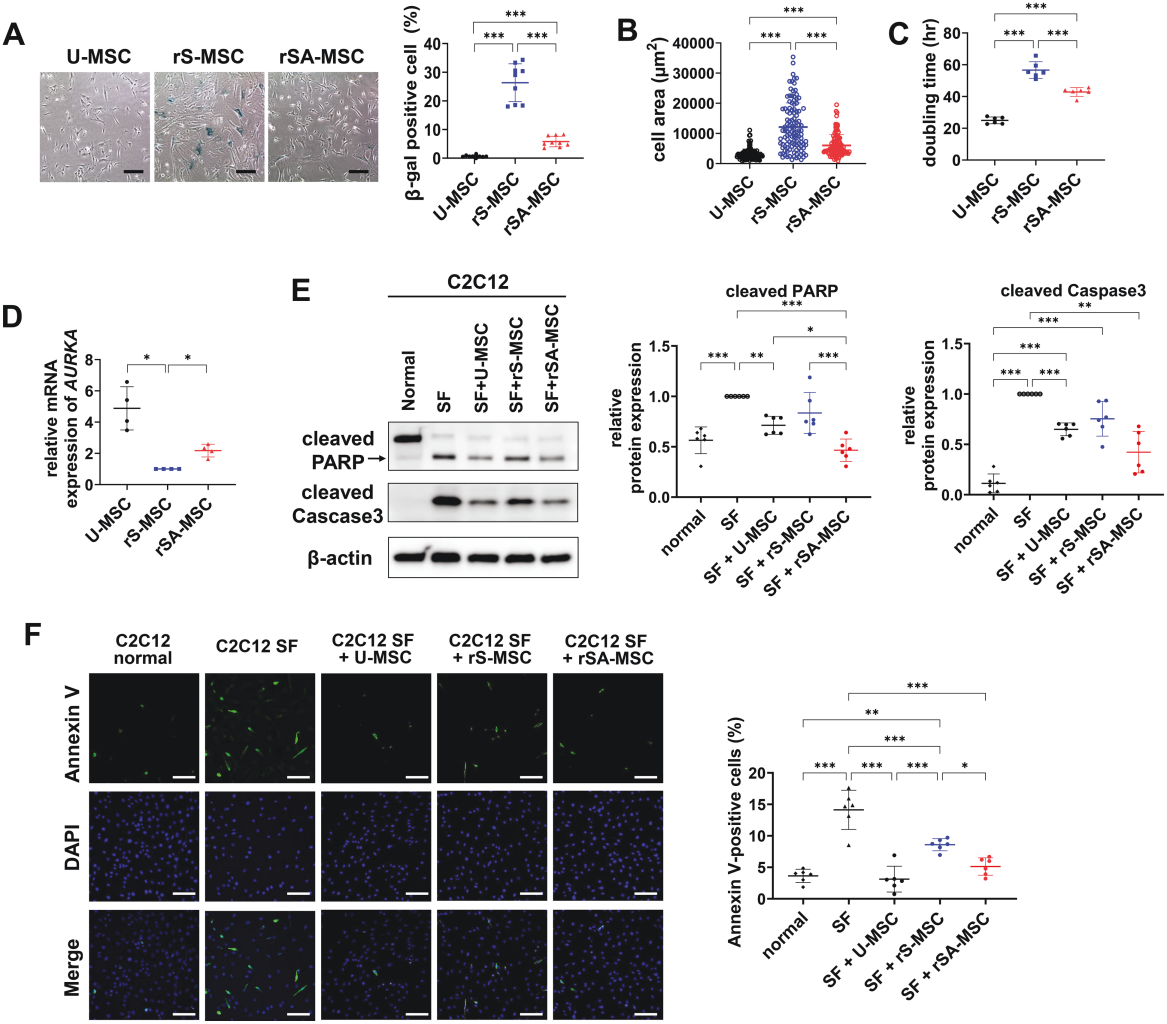
Replicative senescence-alleviated MSCs (rSA-MSCs) were more effective at inhibiting apoptosis in myoblasts than rS-MSC. (A) The proportions of β-gal-positive cells in U-MSCs, rS-MSCs, and rSA-MSCs. Scale bar = 200 µm. (B) Quantification of cell area, (C) doubling time, and (D) mRNA expression levels of aurora kinase A (*AURKA*) in U-MSCs, rS-MSCs, and rSA-MSCs. (E) Immunoblotting using anti-cleaved PARP and anti-cleaved Caspase 3 antibodies and (F) immunocytochemical analysis of annexin V were conducted after coculture of serum-starved C2C12 cells with U-MSCs, rS-MSCs, and rSA-MSCs. Scale bar = 100 µm. The chart was used to quantify the proportion of annexin V-positive cells among the total number of cells. One-way ANOVA with Dunnett’s multiple comparisons post hoc test (A-E) and one-way ANOVA with Tukey’s multiple comparisons post hoc test (C, F) were performed. ***P < .001, **P < .01, and *P < .05. Abbreviation: SF, serum-free.

Replicatively senescent MSCs were treated with BPTES for 24 hours to induce senolysis, and residual cells were recovered after culturing in a normal culture medium for 6 days to obtain replicative senescence-alleviated MSCs (rSA-MSCs; Supplementary Figure S4A). In BPTES-treated group compared to the DMSO-treated group, cell numbers increased significantly on day 6 in normal culture medium, with a decrease in the proportion of β-gal-positive cells and an increase in phosphorylation of ERK (Supplementary Figure S4B-S4D). It was also confirmed that rSA-MSCs had a higher proliferative capacity than that of rS-MSCs even after subculture (Supplementary Figure S4E). Compared with rS-MSCs, the proportion of β-gal-positive cells (Figure 3A), cell area (Figure 3B), and doubling time were remarkably decreased in rSA-MSCs (Figure 3C). Other GLS1 inhibitors also lowered the proportion of β-gal-positive cells among rS-MSCs (Supplementary Figure S5). The expression of *aurora kinase A* (*AURKA*) was higher in rSA-MSCs than in the rS-MSCs (Figure 3D).

AURKA levels and proliferative ability are associated with the anti-apoptotic effects of WJ-MSCs in in vitro and in vivo models of muscle disease.^5^ Therefore, we confirmed the therapeutic effects of rSA-MSCs on skeletal muscles. The anti-apoptotic effect was confirmed by coculturing MSCs with C2C12 cells in which apoptosis had been induced. After inducing apoptosis via serum starvation,^29^ C2C12 cells were cocultured with U-MSCs, rS-MSCs, and rSA-MSCs. The expression levels of apoptosis marker, such as cleaved PARP and cleaved caspase 3 increased in serum-starved C2C12 cells compared to those in normal C2C12 cells, confirming that apoptosis was induced in serum-starved C2C12 cells. The expression levels of apoptosis markers in apoptosis-induced C2C12 cells were not significantly different from those in C2C12 cells cocultured with rS-MSCs, but were remarkably decreased in C2C12 cells cocultured with U-MSCs or rSA-MSCs (Figure 3E).

Annexin V staining was performed to verify the effect of MSCs on apoptosis. The proportion of annexin V-positive cells increased significantly in serum-starved C2C12 cells compared to that in normal C2C12 cells but decreased in C2C12 cells cocultured with MSCs. The proportion of annexin V-positive cells increased considerably in C2C12 cells cocultured with rS-MSCs compared to that in C2C12 cells cocultured with U-MSCs or rSA-MSCs (Figure 3F). These results indicated that BPTES treatment can recover the reduction in therapeutic efficacy by replicative senescence.

### Replicative senescence-alleviated MSCs are effective at the recovery of skeletal muscle in mdx mouse

**Figure 4.**
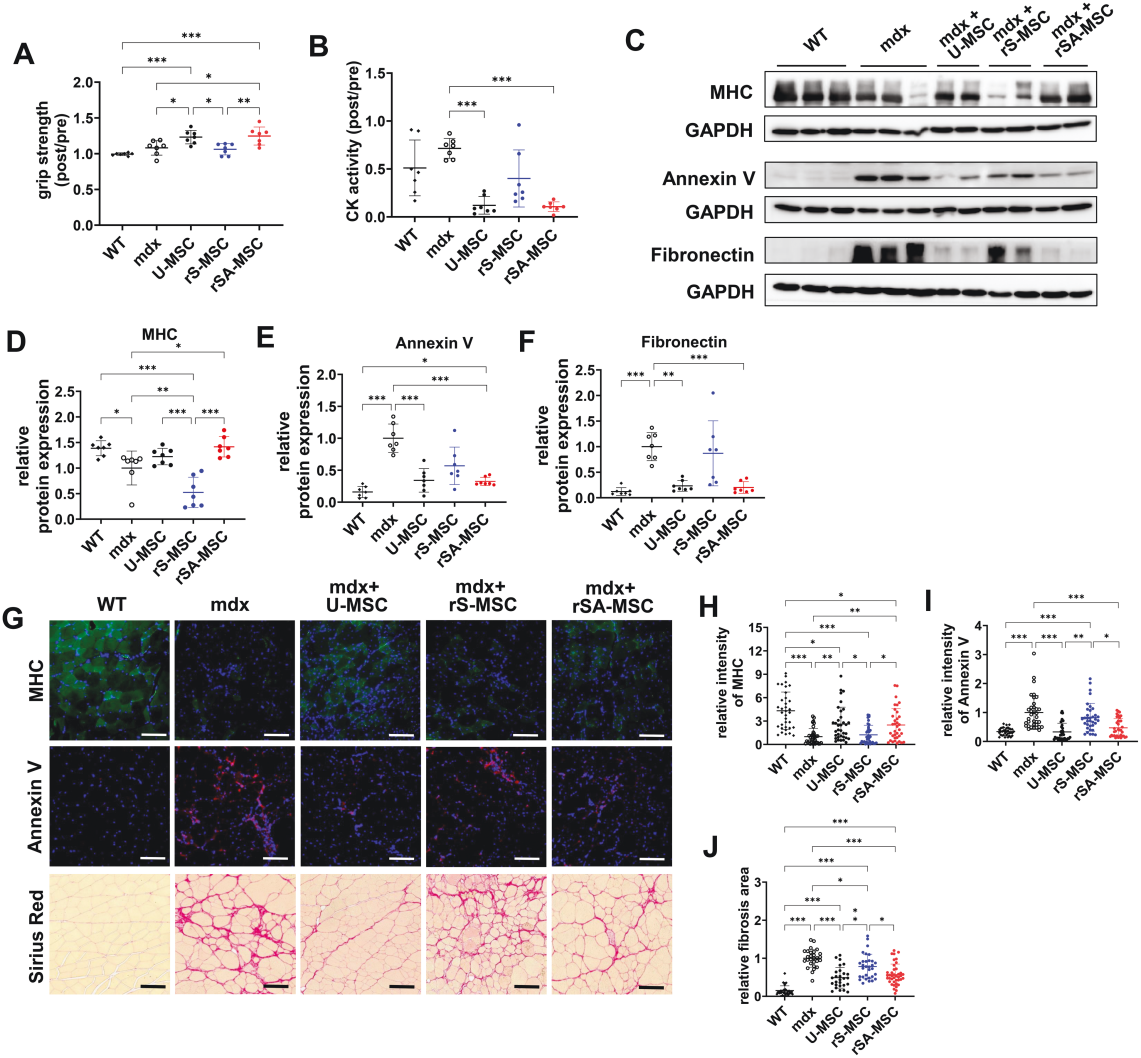
Compared with rS-MSCs, rSA-MSCs were more effective in the recovery of skeletal muscles in mdx mice. (A) Grip strengths of forelimbs and hindlimbs, and (B) creatine kinase (CK) activity were analyzed by comparing preinjection and postinjection in normal, mdx, U-MSC-injected mdx, rS-MSC-injected mdx, and rSA-MSC-injected mdx mice. (C) Immunoblotting was conducted in normal, mdx, U-MSC-injected mdx, rS-MSC-injected mdx, and rSA-MSC-injected mdx mice using the indicated antibodies. Relative protein levels of (D) myosin heavy-chain (MHC), (E) annexin V, and (F) fibronectin indicate the converted value based on that of the mdx group. (G) Immunohistochemical analysis shows immunostaining of MHC and annexin V and Sirius Red staining. Scale bar = 100 µm. The charts were used to quantify the intensities of (H) MHC and (I) annexin V divided by the intensity of DAPI staining. (J) The chart compares the results of the fibrotic area. One-way ANOVA with Tukey’s multiple comparisons post hoc test (A, D) and one-way ANOVA with Dunnett’s multiple comparisons post hoc test (B, E-J) were performed. ***P < .001, **P < .01, and *P < .05.

To verify the therapeutic effects of rSA-MSCs in vivo, U-MSCs, rS-MSCs, and rSA-MSCs were injected into mdx mice (Duchenne muscular dystrophy [DMD] model mice), in which various symptoms of DMD had been confirmed.^4,5^ Grip strength was significantly higher in U-MSC- or rSA-MSC-injected mdx mice than in mdx mice, whereas rS-MSCs did not enhance the grip strength of mdx mice (Figure 4A). CK activity, an indicator of muscle damage, was also significantly reduced after the injection of U-MSCs and rSA-MSCs in mdx mice. However, rS-MSCs did not affect the CK levels in mdx mice (Figure 4B).

An increase in the proportion of mature myofibers was confirmed by MHC expression analysis. Compared to mdx mice, rS-MSC-injected mdx mice exhibited significantly decreased MHC levels, and rSA-MSC-injected mdx mice exhibited significantly increased MHC levels (Figure 4C, 4D). Annexin V levels were significantly increased in the skeletal muscles of mdx mice compared with those in wild-type mice. Injection of U-MSCs or rSA-MSCs reduced annexin V levels in mdx mice, but the injection of rS-MSCs did not significantly reduce annexin V levels in mdx mice (Figure 4C, 4E). The levels of fibronectin, a fibrosis marker, were significantly reduced in U-MSC- and rSA-MSC-injected mdx mice compared to those in mdx mice, whereas no difference in fibronectin levels was observed in rS-MSC-injected mdx mice (Figure 4C, 4F).

Immunohistochemistry revealed that MHC expression was considerably lower in mdx mice than that in wild-type mice. However, with the exception of rS-MSC-injection, the injection of U-MSCs or rSA-MSCs significantly increased MHC expression (Figure 4G, 4H). Conversely, annexin V levels were considerably higher in mdx mice than those in wild-type mice. Although rS-MSCs did not affect the levels of annexin V, U-MSCs and rSA-MSCs reduced annexin V levels in mdx mice (Figure 4G, 4I). Sirius Red staining was conducted to evaluate collagen accumulation, a parameter for the determination of fibrosis. Although fibrosis was significantly enhanced in mdx mice compared to that in wild-type mice, the injection of MSCs reduced collagen accumulation in mdx mice. Especially, U-MSCs and rSA-MSCs were more effective than rS-MSCs at decreasing skeletal muscle fibrosis (Figure 4G, 4J). They also showed remarkable antifibrotic effects in the heart and diaphragm when compared to rS-MSCs (Supplementary Figure S6A, S6B). Taken together, U-MSCs and rSA-MSCs showed excellent therapeutic effects by relieving symptoms in mdx mice. Replicative senescence reduces the therapeutic efficacy of MSCs; however, BPTES treatment improves the therapeutic efficacy by hindering MSC senescence.

### In replicatively senescent MSCs, expression of cathepsin C, E2F transcription factor 7, and sorbin and SH3 domain containing 2 is restored by GLS1 inhibition

**Figure 5.**
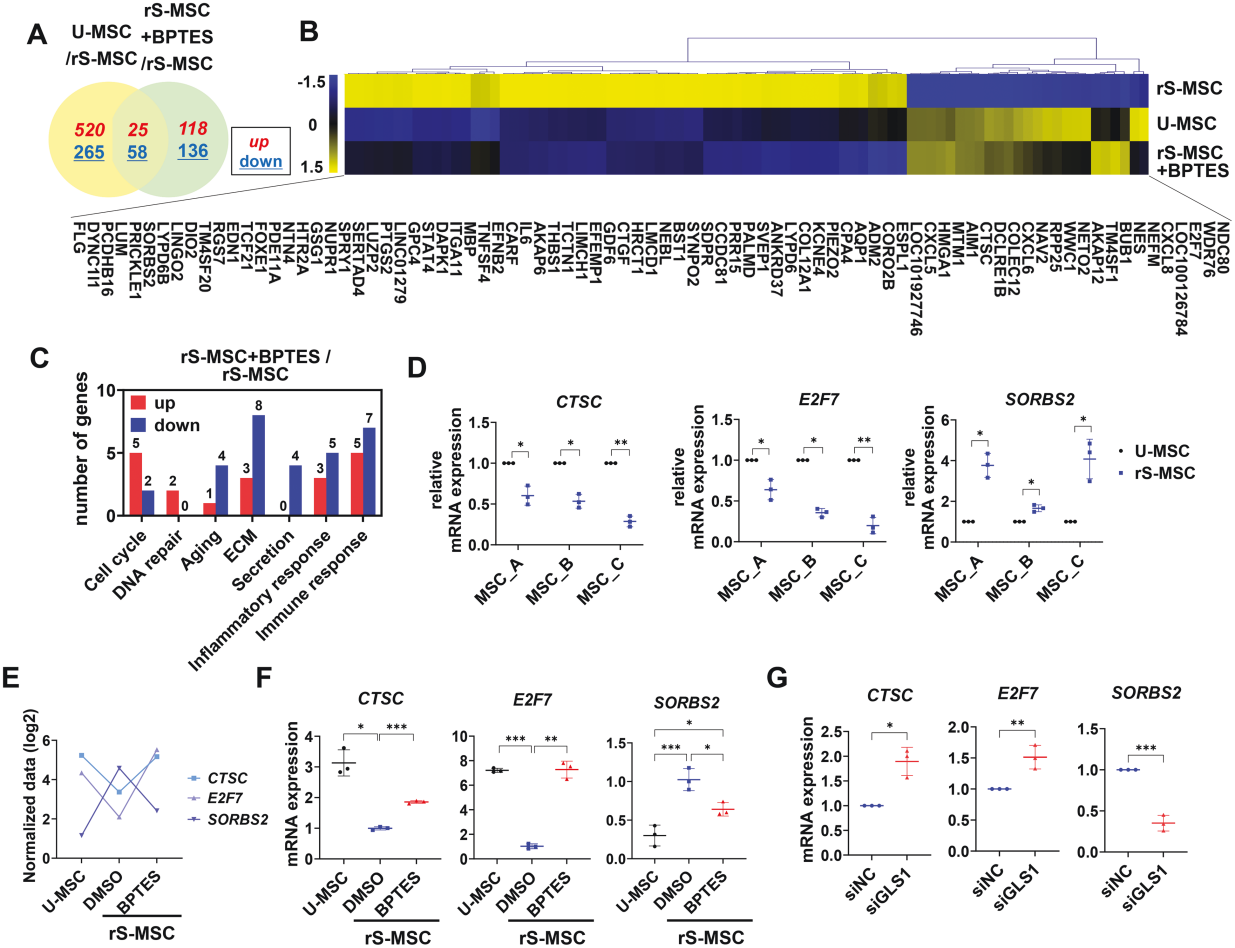
In rS-MSCs, mRNA expression of cathepsin C (*CTSC*), E2F transcription factor 7 (*E2F7*), and sorbin and SH3 domain containing 2 (*SORBS2*) was restored by GLS1 inhibition. (A) Venn diagram displaying the differentially expressed gene (DEG) distribution and (B) heatmap showing DEGs in U-MSCs, rS-MSCs, and BPTES-treated rS-MSCs. (C) Gene categories corresponding to DEGs in rS-MSCs and BPTES-treated rS-MSCs. (D) Levels of *CTSC, E2F7*, and *SORBS2* mRNA in U-MSCs and rS-MSCs. (E) Normalized log2 values of *CTSC, E2F7*, and *SORBS2* in U-MSCs, rS-MSCs, and BPTES-treated rS-MSCs obtained from the mRNA sequencing analysis. (F) Levels of *CTSC*, *E2F7*, and *SORBS2* mRNA in U-MSCs, rS-MSCs, and BPTES-treated rS-MSCs. (G) Levels of *CTSC*, *E2F7*, and *SORBS2* mRNA in rS-MSCs after siGLS1 transfection. Unpaired 2-tailed Student’s *t* test (D, G), one-way ANOVA with Dunnett’s multiple comparisons post hoc test (F; *CTSC* and *E2F7*), and one-way ANOVA with Tukey’s multiple comparisons post hoc test (F; *SORBS2*) were performed. ***P < .001, **P < .01, and *P < .05.

The rS-MSCs that survive BPTES-induced senolysis undergo genetic changes. Thus, we attempted to identify the genetic changes in the remaining MSCs after BPTES treatment and identified senescence-related candidate genes whose expression recovered after BPTES treatment. Differentially expressed genes (DEGs; FC > 3) were identified in U-MSCs, rS-MSCs, and BPTES-treated rS-MSCs. In U-MSCs and BPTES-treated rS-MSCs, 25 genes were upregulated, and 58 genes were downregulated (Figure 5A). These genes were recovered from the replicative senescence following BPTES treatment. A hierarchically clustered heatmap revealed 83 DEGs in U-MSCs, rS-MSCs, and BPTES-treated rS-MSCs (Figure 5B). Compared to untreated rS-MSCs, BPTES-treated rS-MSCs showed a higher number of genes associated with cell cycle and DNA repair as being upregulated, and a higher number of genes associated with aging, extracellular matrix (ECM), secretion, inflammatory response, and immune response as being downregulated (Figure 5C).

We selected genes that were associated with senescence but had not been studied in MSCs by referring to previous studies conducted on the 83 DEGs. The genes cathepsin C (*CTSC*), E2F transcription factor 7 (*E2F7*), and sorbin and SH3 domain containing 2 (*SORBS2*) are involved in senescence,^30-32^ but have not yet been studied in WJ-MSCs. We established rS-MSCs from 3 other donors and confirmed the expression of senescence-related genes using qRT-PCR. In rS-MSCs, *CTSC* and *E2F7* were downregulated, and *SORBS2* was upregulated, compared to that in U-MSCs (Figure 5D). The normalized data validated through qRT-PCR revealed that BPTES treatment restored the expression of these genes (Figure 5E, 5F). Moreover, GLS1 knockdown in rS-MSCs resulted in the same expression pattern as that observed after BPTES treatment (Figure 5G). Therefore, these genes were appropriate senescence gene candidates whose expression was recovered after GLS1 inhibition.

### In replicatively senescent MSCs, GLS1 inhibition alleviates senescence via the Wnt signaling pathway

**Figure 6.**
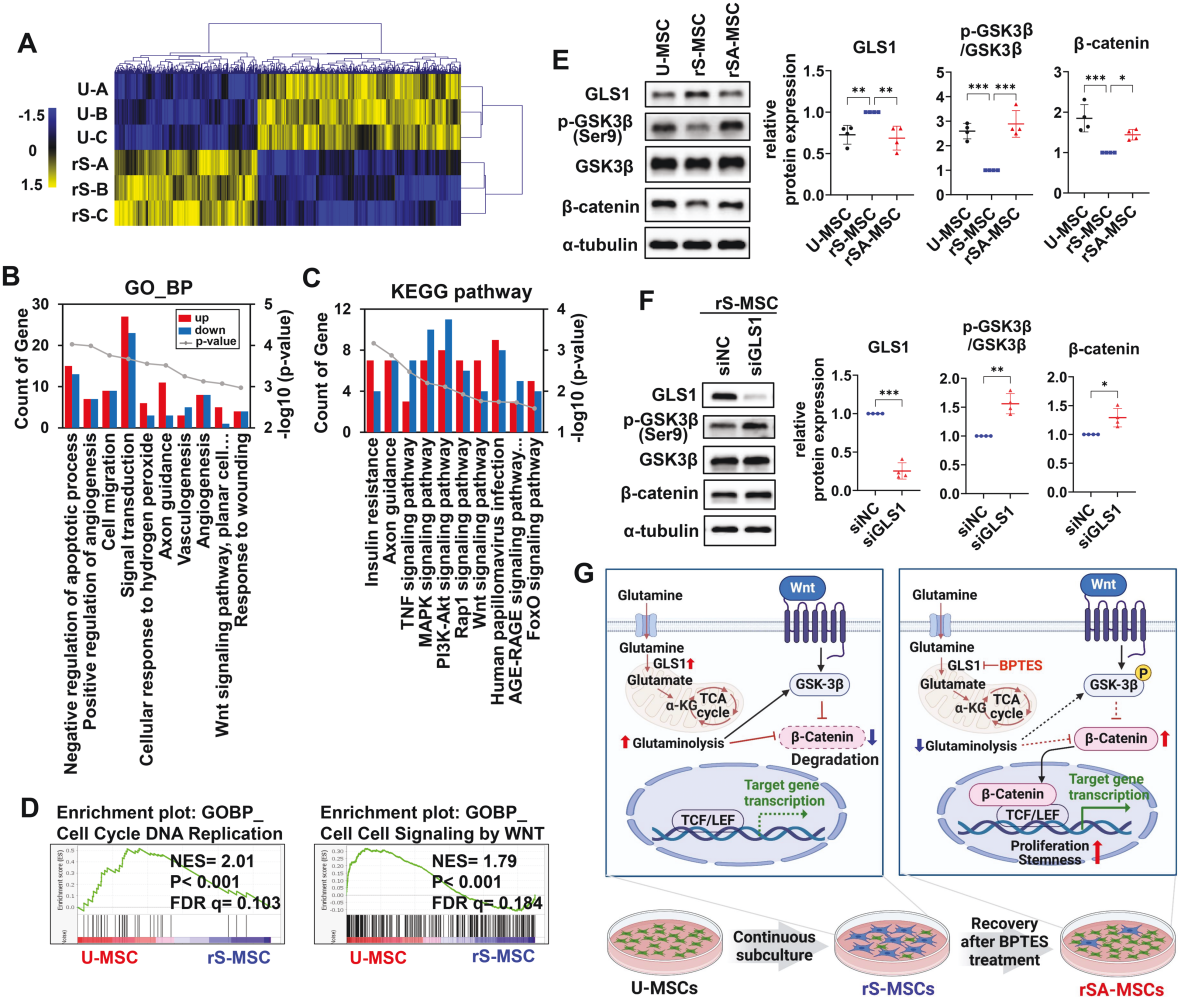
GLS1 participates in hindering senescence by activating Wnt signaling. (A) Heatmap showing the DEGs in U-MSCs and rS-MSCs groups. The functional classification of 545 DEGs based on (B) biological process (BP) of gene ontology (GO) term analysis and (C) Kyoto Encyclopedia of Genes and Genomes (KEGG) pathway analysis. The bars represent the number of DEGs in U-MSCs compared to that in rS-MSCs. (D) Enrichment plots based on gene set enrichment analysis (GSEA) showed that replicative senescence in WJ-MSCs is associated with cell cycle DNA replication and cell-cell signaling by Wnt. (E) Immunoblotting of U-MSCs, rS-MSCs, and rSA-MSCs, and (F) immunoblotting of rS-MSCs after siGLS1 transfection using the indicated antibodies. (G) An increase of GLS1 due to senescence inactivates the Wnt signaling pathway in rS-MSCs, but inhibition of GLS1 by BPTES inhibits replicative senescence by activating the Wnt signaling pathway in rSA-MSCs. One-way ANOVA with Dunnett’s multiple comparisons post hoc test (E) and unpaired 2-tailed Student’s *t* test (F) were performed. ***P < .001, **P < .01, and *P < .05. Abbreviations: NES, normalized enrichment score; FDR, false discovery rate.

The transcriptomes of U-MSCs and rS-MSCs were analyzed to determine the genetic significance of replicative senescence in WJ-MSCs. The hierarchically clustered heatmap showed 320 upregulated and 225 downregulated genes in U-MSCs compared to rS-MSCs (FC > 1.5; *P* < .05; Figure 6A). For functional annotation, DAVID analysis was performed on these DEGs. The *P*-values and number of genes corresponding to each category are indicated. Annotation was performed using the GO and KEGG databases, and the results showed the top 10 categories based on *P*‑values (*P* < .05). Under biological processes (BPs), the GO categories “Wnt signaling pathway, planar cell polarity pathway” (Figure 6B), and “positive regulation of canonical Wnt signaling pathway” were identified (Supplementary Table S3). The DEGs identified through the KEGG pathway were also associated with the “Wnt signaling pathway” (Figure 6C; Supplementary Table S4). Next, GSEA was performed on 11 313 genes to identify significant gene sets related to replicative senescence. GSEA revealed that the gene sets enriched in the U-MSCs included “DNA replication” and “Wnt” (nominal *P* < .05; FDR *q* < 0.25; Figure 6D; Supplementary Table S5). The BP “Wnt signaling pathway” was obtained using DAVID as well as GSEA. Moreover, the levels of β-catenin, a Wnt signaling protein, decreased with senescence (Supplementary Figure S7A). This suggests that the Wnt signaling pathway is associated with replicative senescence in WJ-MSCs.

The expression of Wnt signaling-related proteins in rSA-MSCs was examined to determine whether the activation of Wnt signaling was associated with the mitigation of senescence. The levels of β-catenin and phosphorylated GSK3β (ser9) were reduced in response to senescence and recovered with a decrease in GLS1 levels in rSA-MSCs (Figure 6E). Similarly, the Wnt signaling pathway in rS-MSCs was activated in response to GLS1-knockdown (Figure 6F) and treatment with other GLS1 inhibitors (Supplementary Figure S7B). These results suggest that an increase in GLS1 expression due to senescence inactivates the Wnt signaling pathway, but inhibition of GLS1 hinders replicative senescence via activation of the Wnt signaling pathway in WJ-MSCs (Figure 6G).

Meanwhile, the gene sets enriched in rS-MSCs correlated with autophagy including “positive regulation of autophagy,” “positive regulation of macroautophagy,” and “macroautophagy” (Supplementary Figure S8A and Supplementary Table S6). Autophagy was confirmed based on the expression of LC3II, which was increased in rS-MSCs compared to U-MSCs, but decreased in rSA-MSCs (Supplementary Figure S8B). GLS1 knockdown and GLS1 inhibitors also reduced LC3II levels in rS-MSCs (Supplementary Figure S8C, S8D). These results imply that autophagy is related to senescence in WJ-MSCs, and that GLS1 may modulate autophagy.

## Discussion

MSC senescence reduces the therapeutic efficacy and impairs the safety of MSCs as therapeutic agents.^7^ Our results showed a significant increase in GLS1 expression in rs-MSCs and demonstrated that the regulation of GLS1 expression by BPTES increased the therapeutic effect of MSCs through senolysis. This is the first study to confirm an association between MSC senescence and GLS1 expression.

Cellular senescence is mediated by the p53/p21 and p16 pathways, which produce SASP and increase SA-β-gal activity.^33^ In particular, SASP proteins secreted by senescent cells induce senescence in the surrounding young cells.^16,19^ Therefore, the removal of senescent cells through senolysis can prevent further senescence induced in response to SASP secretion. In our study, GLS1 inhibition caused senolysis of WJ-MSCs and reduced the expression of p16, p21, and SASP factors. In addition, BPTES treatment in early passage considerably delayed senescence during repeated subcultures (Supplementary Figure S9). Furthermore, BPTES mitigated replicative senescence of BM-MSCs (Supplementary Figure S10). Based on these results, we suggest that GLS1 may be a target for regulating the replicative senescence of MSCs.

In a previous study, we demonstrated that the expression of AURKA is donor-dependent and associated with the proliferative capacity and therapeutic efficacy of WJ-MSCs, because AURKA can be used as a marker for the proliferation and therapeutic efficacy of WJ-MSCs.^5^ In this study, we observed that the replicatively senescent WJ-MSCs showed decreased *AURKA* levels, which reduced the therapeutic effect of WJ-MSCs. However, rSA-MSCs recovered after treatment with BPTES showed increased *AURKA* expression and improved therapeutic effects in in vitro and in vivo muscle disease model compared to rS-MSCs. Patients with DMD have severely degenerated myofibers in skeletal muscles, but ultimately lead to death due to cardiorespiratory dysfunction.^34^ U-MSCs and rSA-MSCs are expected to have therapeutic effects on cardiorespiratory dysfunction as they reduced fibrosis of the heart and diaphragm as well as skeletal muscle. Collectively, these findings suggest that GLS1 can also serve as a target for regulating the therapeutic effects of MSCs, and that inhibition of GLS1 may enhance the therapeutic efficacy of senescent MSCs.

We sought to identify the target genes affected by GLS1 inhibition to understand the mechanism underlying the regulation of replicative senescence via senolysis. Among these target genes, *CTSC*,^30^*SORBS2*,^31^ and *E2F7*^32^ have been reported to be related to senescence. Specifically, CTSC is a lysosomal cysteine protease, and its knockdown inhibits cell proliferation and enhances senescence in tumor cells.^30^ Endogenous level of SORBS2 increase with keratinocyte senescence,^31^ and E2F7 accelerates oncogene-induced senescence.^32^ Our results showed that the expression of these genes was dependent on the replicative senescence of WJ-MSCs and GLS1 inhibition. However, an association between these genes and GLS1 inhibition in MSCs has not yet been reported. Therefore, we expect that further studies on the roles of these genes in the replicative senescence of MSCs will provide insights into the senescence-related mechanisms.

Next, we investigated the signaling pathways associated with senescence in WJ-MSCs through transcriptome analysis. The Wnt signaling pathway was confirmed to be associated with replicative senescence in WJ-MSCs. Recent studies have reported that Wnt/β-catenin signaling impairs senescence by inhibiting the secretion of SASP factors in human bone marrow-derived MSCs,^35^ whereas noncanonical Wnt signaling induces senescence via the JAK-STAT pathway in tendon stem/progenitor cells.^36^ β-Catenin, the main mediator of the Wnt pathway, is degraded by a multi-protein complex containing GSK3β,^37^ and phosphorylation of GSK3β at ser9 inhibits GSK3β activity.^38^ Our results indicate that the replicative senescence of WJ-MSCs caused a decrease in the β-catenin levels; conversely, inhibition of GLS1 in senescent MSCs promoted the phosphorylation of GSK3β at the Ser9 residue and increased the expression of β-catenin. Therefore, the Wnt signaling pathway is potentially associated with the regulation of senescence in WJ-MSCs via GLS1.

The paracrine factors secreted from MSCs contribute to therapeutic effects in various diseases.^3^ Among paracrine factors verified through transcriptome analysis, CXCL6 content was increased in rSA-MSCs via GLS1 inhibition (Supplementary Figure S11). CXCL6 can be helpful to the therapy of acute myeloid leukemia by improving arterial niche through the promotion of angiogenesis in human umbilical artery endothelial cells (HUAECs) and the reduction of apoptosis in MSCs and HUAEC.^39^ Furthermore, CXCL6 was upregulated by β-catenin.^40^ In rS-MSCs, GLS1 inhibition increased both β-catenin and CXCL6. Although further experiments are required, the enhancement of the therapeutic effect of rSA-MSC on muscle disease might have been affected by the upregulation of CXCL6 due to increased β-catenin.

Autophagy, which is essential for homeostasis, can not only inhibit senescence, but can also promote SASP synthesis and induce senescence.^41^ Autophagy mediated by p38α promoted senescence in cancer cells, which confirmed that LC3II, an autophagy marker, was upregulated in senescent cells.^42^ We confirmed that LC3II levels were increased during senescence and decreased after treatment with GLS1 inhibitors and GLS1 knockdown in WJ-MSCs. The results indicate that GLS1 is involved in autophagy in senescent WJ-MSCs. However, glutaminolysis contributed to autophagy in cancer cells,^21,43^ which is contrary to our results and may be due to differences in cell type or cellular senescence. Meanwhile, as autophagy has been reported to negatively regulate Wnt signaling,^44,45^ further research on the association between autophagy and Wnt signaling in WJ-MSCs is required.

Among the various GLS1 inhibitors reported previously,^26^ we selected BPTES, which is the most potent chemical for inducing senolysis and inhibiting senescence. Previous studies have shown that continuous BPTES treatment eliminates senescent fibroblasts and mitigates their senescent phenotype in vivo.^22,23^ In WJ-MSCs, BPTES eliminated senescent cells through senolysis and inhibited senescence. However, unlike that in previous studies, we confirmed that the therapeutic efficacy of replicatively senescent WJ-MSCs was improved by providing a sufficient recovery period after short-term BPTES treatment. Thus, we established the optimal conditions for BPTES treatment to enhance the therapeutic effect of MSCs. This study is expected to contribute to improving the therapeutic effect of senescent MSCs and increase our understanding of the replicative senescence of MSCs. However, a bottleneck that needs to be overcome is that BPTES is not a clinically available drug owing to its low water solubility and pharmacokinetic properties.^26,46^ In addition, the toxicity of solvents in which BPTES can be dissolved is also a factor that makes clinical application difficult.^47^ As GLS1 can be a target for controlling replicative senescence, the determination of suitable treatment conditions or appropriate inhibitors that can be used clinically is important to minimize toxicity.

## Conclusion

To our knowledge, this is the first study to confirm that GLS1 is a key factor in modulating replicative senescence in MSCs. Furthermore, MSCs senescence weakens the therapeutic effect of MSCs, and GLS1 inhibitors such as BPTES can be used to restore the therapeutic efficacy of senescent MSCs. Therefore, we suggest that GLS1 regulation is a potential strategy for controlling the senescence of MSCs via senolysis, which can increase the success rate of clinical trials by enhancing the proliferation and therapeutic potential of MSCs.

## Supplementary Material

Supplementary material is available at *Stem Cells Translational Medicine* online.

szae053_suppl_Supplementary_Tables_S1-S6_Figures_S1-S11

## Data Availability

The data used to support the findings of this study are available from the corresponding authors upon request.
