# Supplementary material for: Glutaminase-1 inhibition alleviates senescence of Wharton’s jelly-derived mesenchymal stem cells via senolysis
Source: Stem Cells Transl Med. 2024 Aug 9;13(9):873–85. doi: 10.1093/stcltm/szae053 (PMC11386220; doi:10.1093/stcltm/szae053)
Supplement: szae053_suppl_Supplementary_Tables_S1-S6_Figures_S1-S11 [file szae053_suppl_supplementary_tables_s1-s6_figures_s1-s11.pdf]

Supplemental Information

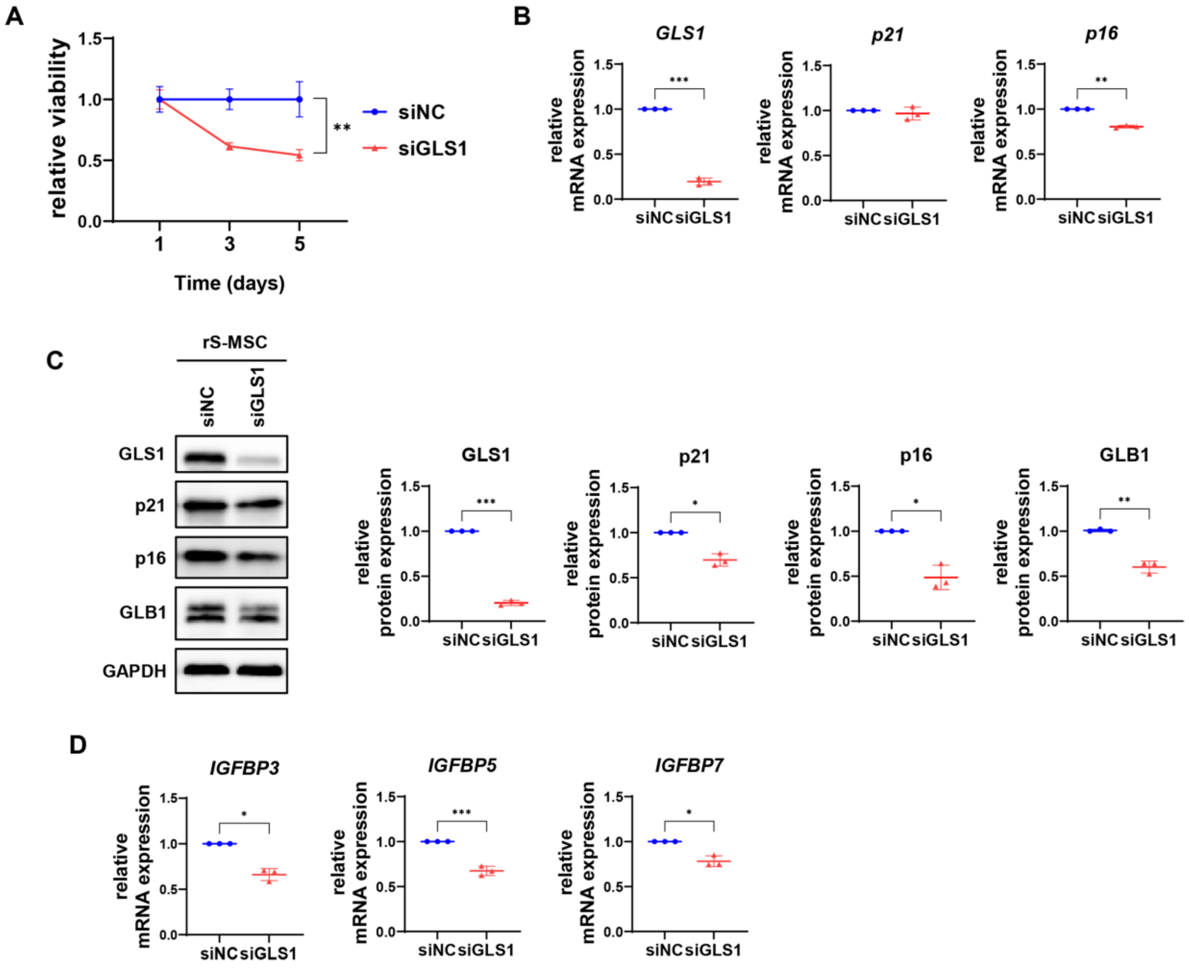

Supplementary Fig. S1. In replicatively senescent mesenchymal stem cells (rS-MSCs), knockdown of glutaminase-1 (GLS1) reduced the expression levels of aging markers. (A) Relative viability of rS-MSCs after small interfering RNA (siRNA) transfection. (B) Levels of *GLS1*, *p21*, and *p16* mRNA, (C) levels of GLS1, p21, p16, and beta-galactosidase-1 (GLB1) protein, and (D) levels of *insulin-like growth factor binding protein 3* (*IGFBP3*), *IGFBP5*, and *IGFBP7* mRNA in rS-MSCs after siRNA transfection. Two-tailed Student's *t*-test (A to D) was performed. ***p < 0.001, **p < 0.01, and *p < 0.05.

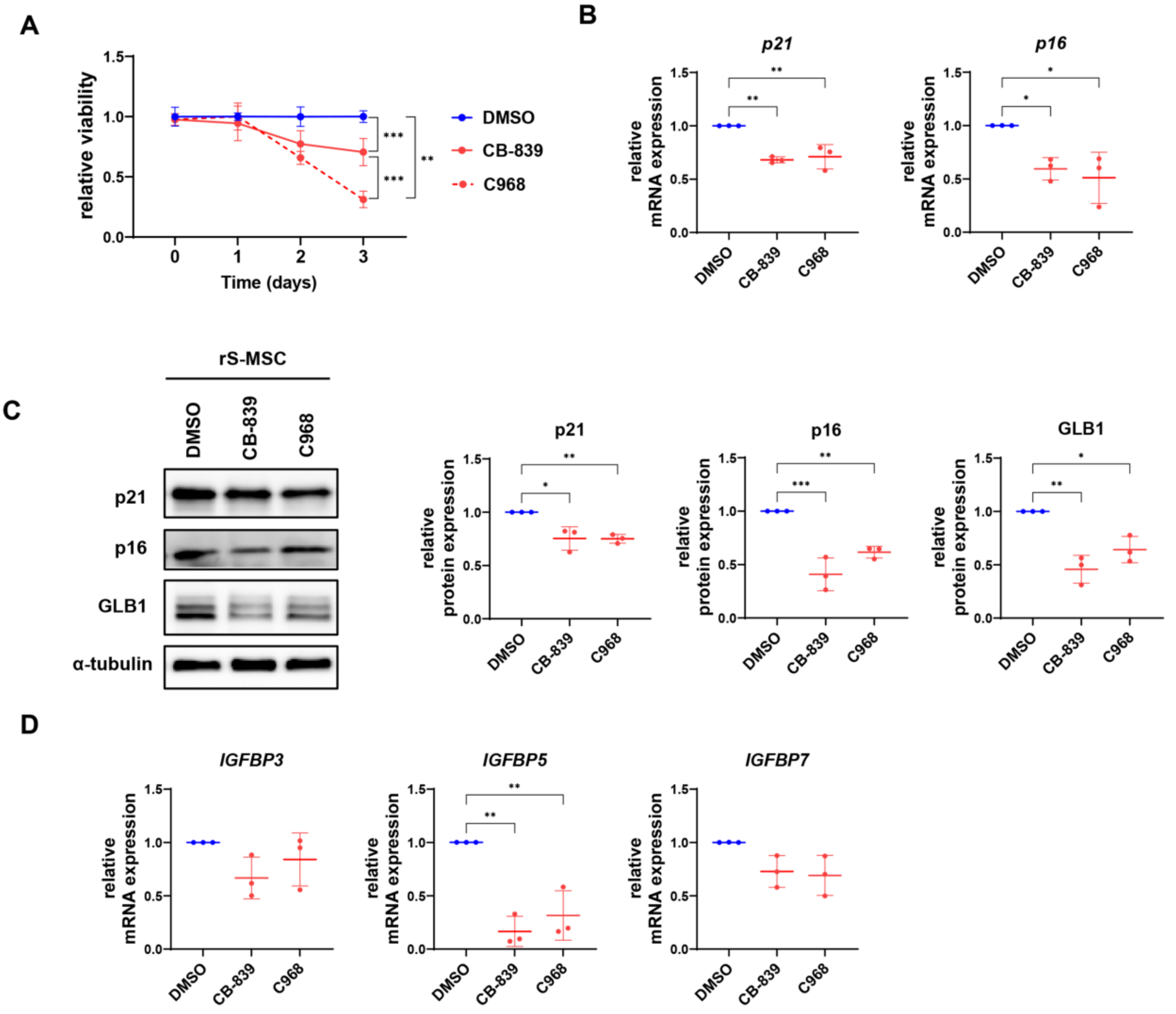

Supplementary Fig. S2. In rS-MSCs, CB-839 and C968 reduced the expression levels of aging markers. (A) Relative viability of rS-MSCs treated with dimethyl sulfoxide (DMSO), CB-839 (1 µM), and C968 (10 µM). (B) Levels of *p21* and *p16* mRNA, (C) levels of p21, p16, and GLB1 protein, and (D) levels of *IGFBP3, IGFBP5*, and *IGFBP7* mRNA in rS-MSCs treated with DMSO, CB-839, and C968. One-way analysis of variance (ANOVA) with Tukey's multiple comparisons post hoc test (A to D) were performed. ***$p < 0.001$, **$p < 0.01$, and *$p < 0.05$.

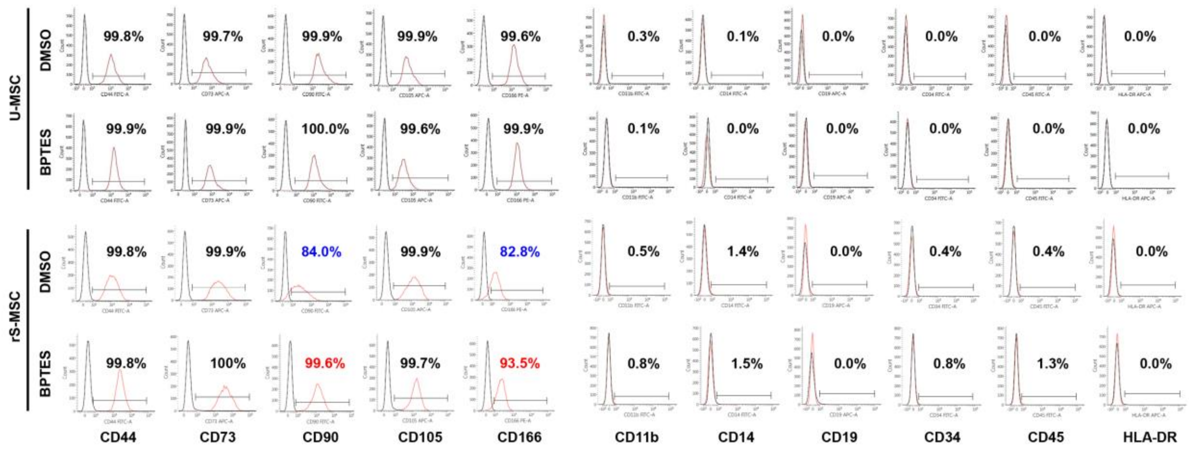

Supplementary Fig. S3. Bis-2-(5-phenylacetamido-1,2,4-thiadiazol-2-yl)ethyl sulfide 3 (BPTES) treatment increased the stemness of rS-MSCs. Stemness of senescence-uninduced MSCs (U-MSCs) and rS-MSCs in the presence or absence of BPTES (30 μM). Stemness was confirmed by conducting fluorescence-activated cell sorting (FACS) using identity markers CD44, CD73, CD90, and CD105 and purity markers CD11b, CD14, CD19 CD34, CD45, and HLA-DR.

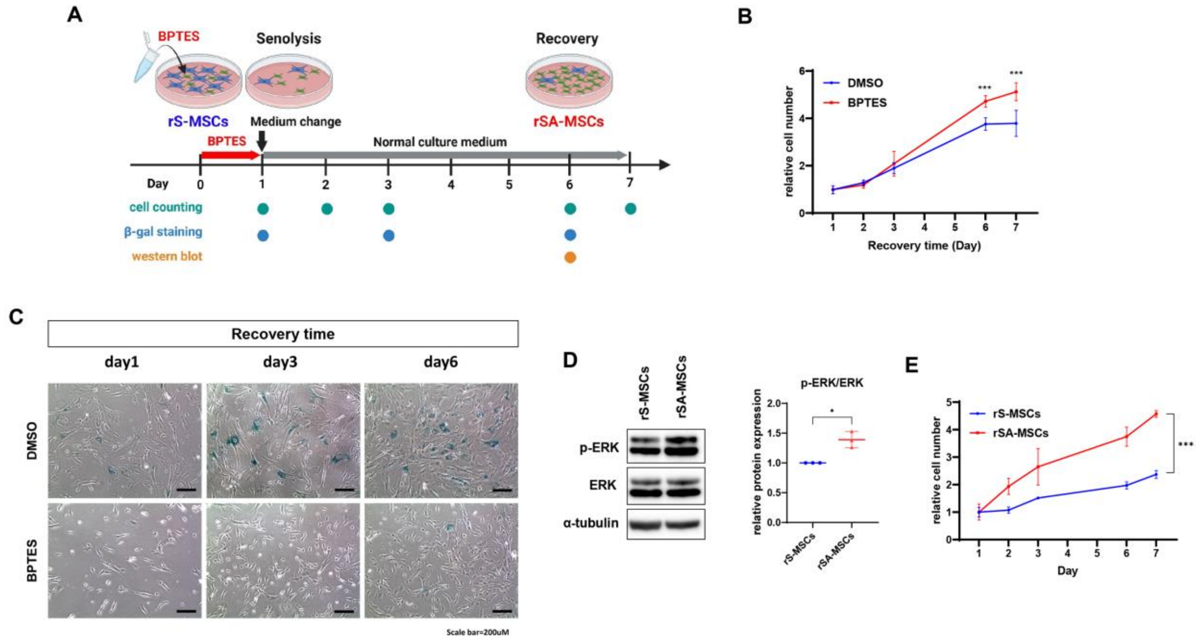

Supplementary Fig. S4. Determination of the optimal conditions for the recovery period from rS-MSCs to rSA-MSCs. (A) Graphical experimental design showing the recovery strategy from rS-MSCs to rSA-MSCs. (B) Cell numbers were confirmed at day 1, 2, 3, 6 and 7 after removal of BPTES. (C) The β-gal staining was performed at day 1, 3 and 6. Scale bar, 200 μm. (D) Immunoblotting using anti-ERK and anti-phospho-ERK antibodies. (E) Cell numbers were counted at day 1, 2, 3, 6 and 7 after subculture. Two-tailed Student's *t*-test (B, D and E) was performed. ***$p < 0.001$ and *$p < 0.05$.

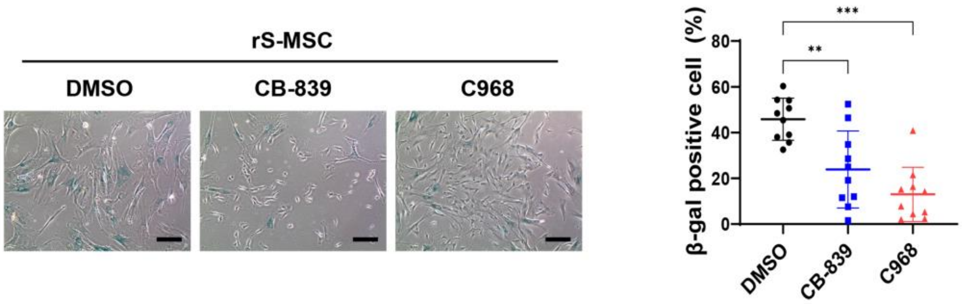

Supplementary Fig. S5. In rS-MSCs, CB-839 and C968 reduced the proportion of β-gal-positive cells. The β-gal-positive cell rate in rS-MSCs treated with DMSO, CB-839 (1 μM), or C968 (10 μM). Scale bar, 200 μm. One-way ANOVA with Tukey's multiple comparisons post-hoc test was performed. ***$p < 0.001$ and **$p < 0.01$.

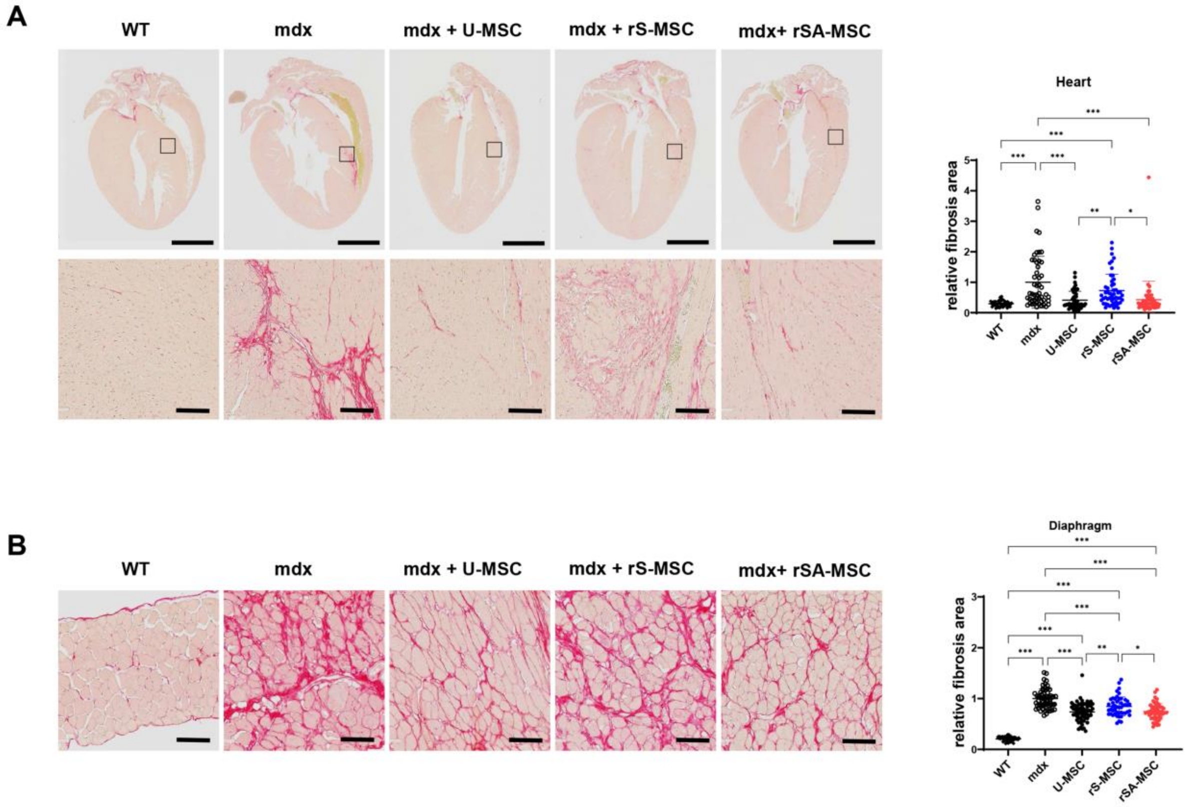

Supplementary Fig. S6. Compared to rS-MSCs, rSA-MSCs had more anti-fibrotic effects in heart and diaphragm of mdx mouse. Sirius Red staining was conducted in normal, mdx, U-MSC-injected mdx, rS-MSC-injected mdx, and rSA-MSC-injected mdx mice. (A) Sirius Red staining of hearts. Scale bar (up), 2mm. Scale bar (down), 100 µm. (B) Sirius Red staining of diaphragms. Scale bar, 100 µm. The chart compares results of the fibrotic area. One-way ANOVA with Tukey's multiple comparisons post-hoc test was performed. ***$p < 0.001$, **$p < 0.01$ and *$p < 0.05$.

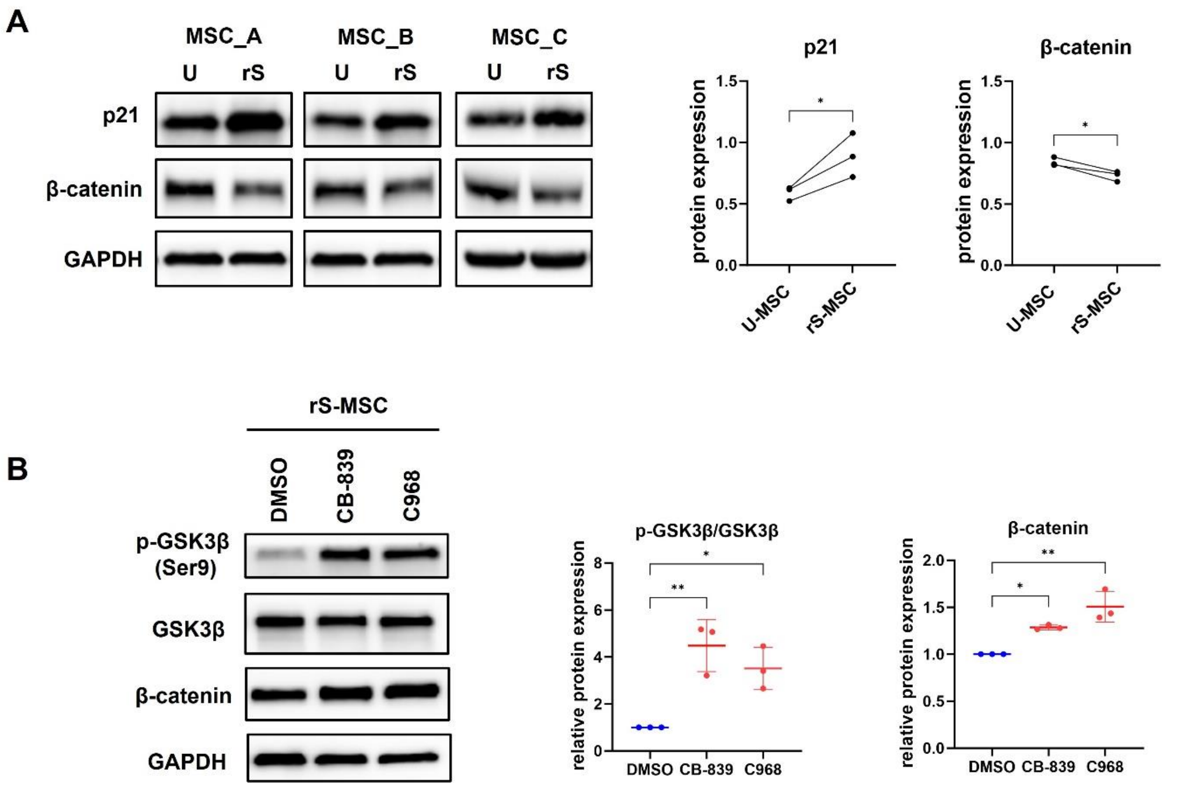

Supplementary Fig. S7. In rS-MSCs, CB-839 and C968 activate Wnt signaling. (A) Immunoblotting of U-MSC and rS-MSC groups. (B) Immunoblotting of rS-MSCs treated with DMSO, CB-839 (1 μM) and C968 (10 μM) using the indicated antibodies. Two-tailed Student's *t*-test (A) and one-way ANOVA with Tukey's multiple comparisons post-hoc test (B) were performed. **$p < 0.01$, and *$p < 0.05$.

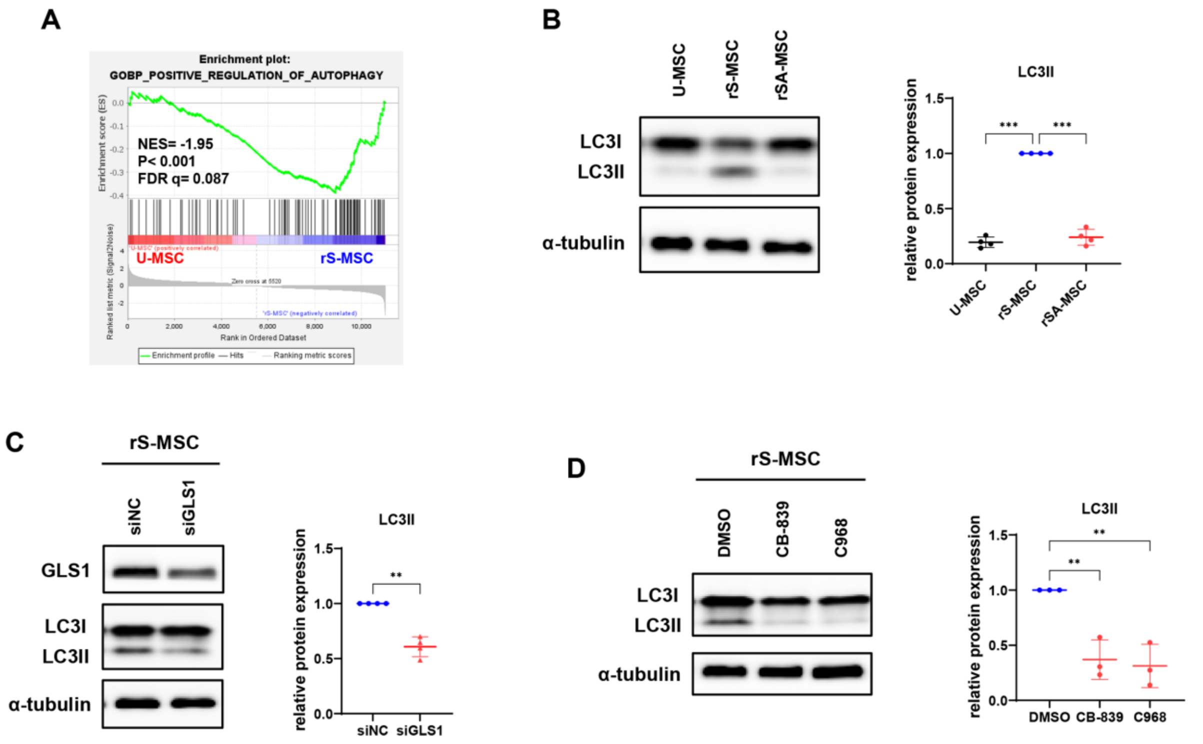

Supplementary Fig. S8. GLS1 inhibition reversed the induction of autophagy by replicative senescence. (A) Enrichment plots corresponding to gene sets enriched in rS-MSCs. (B) Immunoblotting of LC3B in U-MSCs, rS-MSCs, and replicative senescence-alleviated MSCs (rSA-MSCs). (C) Immunoblotting of LC3B in rS-MSCs after siGLS1 transfection. (D) Immunoblotting of LC3B in rS-MSCs treated with DMSO, CB-839 (1 μM), or C968 (10 μM). One-way ANOVA with Tukey's multiple comparisons post-hoc test (B and D) and two-tailed Student's *t*-test (C) were performed. ***$p < 0.001$, **$p < 0.01$, and *$p < 0.05$.

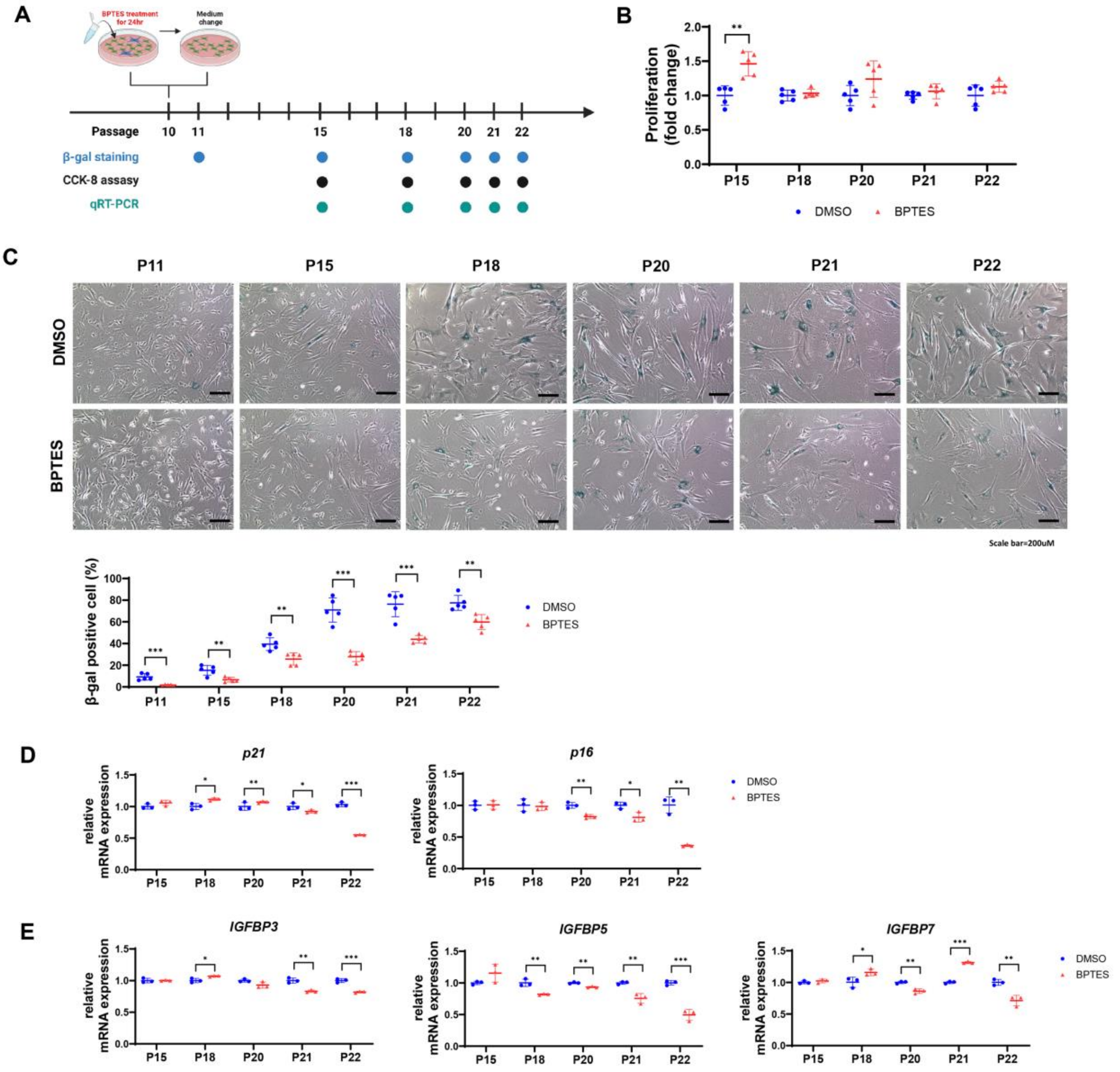

Supplementary Fig. S9. BPTES treatment in the early passage delayed replicative senescence. (A) Illustration of the experimental procedure. (B) Proliferation was calculated with the OD values of BPTES group compared to the OD values of DMSO group. (C) The β-gal staining was performed until passage 22. Scale bar, 200 μm. (D) mRNA levels of *p21*and *p16*, and (E) mRNA levels of *IGFBP3*, *IGFBP5*, and *IGFBP7* in DMSO group and BPTES group. Two-tailed Student's *t*-test (B to E) was performed. ***$p < 0.001$, **$p < 0.01$, and *$p < 0.05$.

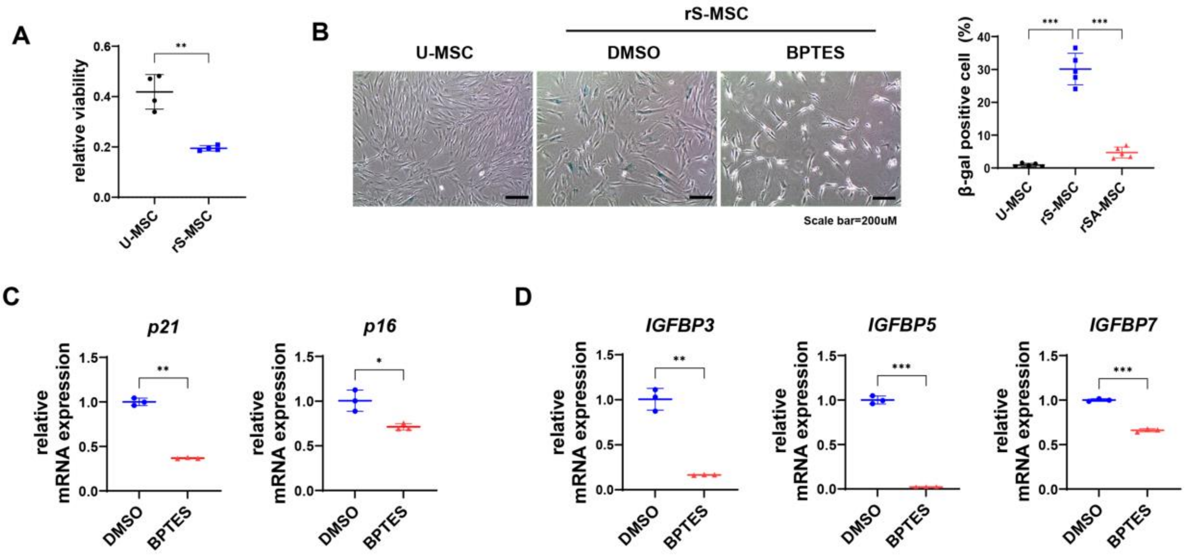

Supplementary Fig. S10. BPTES alleviated replicative senescence in bone marrow-derived MSCs. (A) Relative viability was calculated with the OD values of BPTES group compared to the OD values of DMSO group in U-MSCs and rS-MSCs. Cell viability was measured after treating with DMSO or BPTES (10 μM) for 72 h. (B) The proportion of β-gal-positive cells was decreased by BPTES in rS-MSCs. Scale bar, 200 μm. (C) mRNA levels of *p21* and *p16*, and (D) mRNA levels of *IGFBP3*, *IGFBP5*, and *IGFBP7* in rS-MSCs treated with DMSO and BPTES. Two-tailed Student's *t*-test was performed. ***$p < 0.001$, **$p < 0.01$ and *$p < 0.05$.

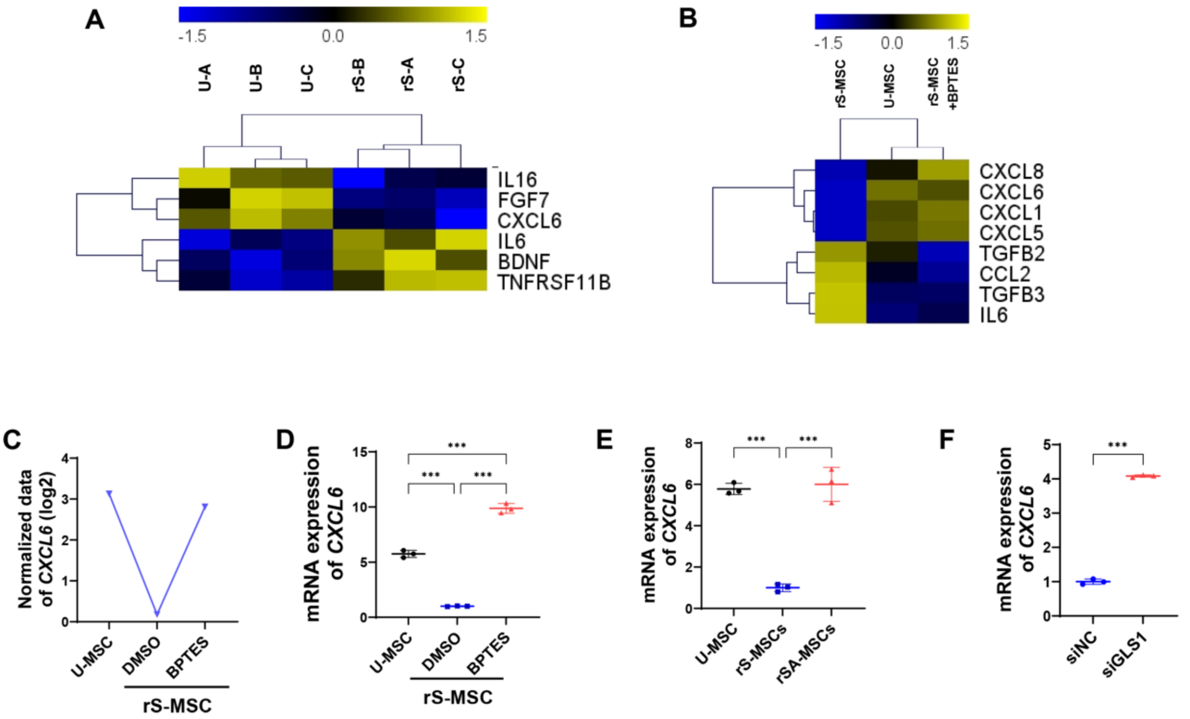

Supplementary Fig. S11. CXCL6 increased in rSA-MSCs by GLS1 inhibition. (A) Heatmap showing DEGs in U-MSCs and rS-MSCs (FC > 2, $p < 0.05$). (B) Heatmap showing DEGs in U-MSCs, rS-MSCs, and BPTES-treated rS-MSCs (FC > 2). (C) Normalized log2 values of *CXCL6*. (D) Level of *CXCL6* in U-MSCs, rS-MSCs, and BPTES-treated rS-MSCs. (E) Level of *CXCL6* in U-MSCs, rS-MSCs, and rSA-MSCs. (F) Level of *CXCL6* in rS-MSCs after siRNA transfection. One-way ANOVA with Tukey's multiple comparisons post-hoc test (D and E) and two-tailed Student's *t*-test (F) were performed. ***$p < 0.001$.

Supplementary Table S1. Small interfering RNAs (siRNAs) with sequences.

Target	siRNA Sequence (5' --> 3')
GLS1_1	Sense) CACGAUCUUUCUCUGU
	Antisense) ACAGAGAAACAAGAUCGUG
GLS1_2	Sense) GAAUAUGUGCAUCGAUAUG
	Antisense) CAUAUCGAUGCACAUAUUC

Supplementary Table S2. Primers used for qRT-PCR

Target	Sequence (5' --> 3')
GAPDH_F	GAAGGTGAAGGTCGGAGT
GAPDH_R	TGGCAACAATATCCACTTTACCA
GLS1_F	CAGAAGGCACAGACATGGTTGG
GLS1_R	GGCAGAAACCACCATTAGCCAG
p16_F	CCCAACGCACCGAATAGTTA
p16_R	ACCAGCGTGTCCAGGAAG
p21_F	AGGTGGACCTGGAGACTCTCAG
p21_R	TCCTCTTGGAGAAGATCAGCCG
IGFBP3_F	AGCTCCAGGAAATGCTAGTG
IGFBP3_R	AACTTGGGATCAGACACCC
IGFBP5_F	GAGCAAGTCAAGATCGAGAG
IGFBP5_R	CTTCTTCACTGCTTCAGCC
IGFBP7_F	CAAAGGACAGAACTCCTGC
IGFBP7_R	TAGAGGAGATACCAGCACC
AURKA_F	GGCCACTGAATAACACCCAAA
AURKA_R	AGAGGGCGACCAATTTCAAAG
CTSC_F	CCTATCTTGACCTGCTGGG
CTSC_R	CTTGTGGTCCCATAACCGA
E2F7_F	CTCGCTATCCAAGTTATCCC
E2F7_R	TTTCCACACCAAGACTGAC
SORBS2_F	CAATGCAGGTCTGTACAACC
SORBS2_R	TTTGGAAAGAGGTCTGTAGGT
CXCL6_F	CGTTACGCTGAGAGTAAACC
CXCL6_R	GTTCTTCAGGGAGGCTACC

Supplementary Table S3. Gene ontology biological process (GOBP) analyzed using DAVID (Top30)

No.	Term	Count	%	p-value	Genes	Fold Enrichment
1	GO:0043066~ negative regulation of apoptotic process	28	5.47	9.40E-05	CITED2, GATA6, SCX, FMN2, THBS1, FOXO1, AQP1, CCND2, CHST11, PDPN, RPS6KA1, ADORA1, PIM1, ANXA1, MSX2, TSLP, MGMT, SPHK1, WNT5A, DHCR24, GREM1, IL6, RASA1, WNK3, PDE3A, FAS, CD44, BCL2L2	2.311
2	GO:0045766~ positive regulation of angiogenesis	14	2.73	1.03E-04	CCBE1, ECM1, FLT1, TNFSF12, SPHK1, ITGB2, WNT5A, GATA6, THBS1, HK2, AQP1, GREM1, ADAM12, CYP1B1	3.734
3	GO:0016477~ cell migration	18	3.52	1.75E-04	LAMA5, FLT1, FOXE1, ITGB2, NTN4, FMN2, RND3, THBS1, SORL1, TGFBR3, BAMBI, PDPN, SPATA13, GPC3, RHOU, HES1, GPC4, CD44	2.905
4	GO:0007165~ signal transduction	50	9.77	2.12E-04	CXCL6, ECM1, IRS1, LRRC2, FGF7, ALCAM, PLAU, RPS6KA1, ADORA1, GRB10, APPL2, MAGI1, GPR39, TLE3, ANXA1, TLE1, KSR1, PRKCE, IL16, GREM1, ITPKB, RASA1, ROR2, PLCB1, OPTN,	1.725

					CARD11, TNFRSF21, HBEGF, SHC3, EPAS1, NPR3, NRXN3, TNFRSF11B, NDRG2, RASIP1, PDE11A, CYTL1, CRADD, TSLP, RASL10B, STAT1, TNFSF12, KCNIP3, MCC, GNG11, TNFSF4, PDE3A, NOSTRIN, FAS, IL7R	
5	GO:0070301~ cellular response to hydrogen peroxide	9	1.76	2.78E-04	IL6, ANXA1, SPHK1, CYP1B1, KLF4, FOXO1, MAPK13, AQP1, MAP3K5	5.320
6	GO:0007411~ axon guidance	14	2.73	3.05E-04	LAMA5, SEMA3D, UNC5B, BDNF, SEMA6D, WNT5A, NRXN3, ETV1, EFNB1, EFNB3, FLRT3, FEZ1, SLIT3, EMB	3.346
7	GO:0001570~ vasculogenesis	8	1.56	5.64E-04	TGFBR3, EGFL7, CITED2, RASA1, CAV1, HAS2, HOXA13, RASIP1	5.554
8	GO:0001525~ angiogenesis	16	3.13	7.45E-04	ECM1, EGFL7, FLT1, UNC5B, EPAS1, TNFSF12, CAV1, NRXN3, VASH1, PTGS2, RASIP1, TGFBR3, KLF5, FAP, HAND2, CYP1B1	2.766
9	**GO:0060071~Wnt signaling pathway, planar cell polarity pathway**	6	1.17	8.46E-04	VANGL2, WNT5A, GPC3, ROR1, ROR2, GPC4	7.953

10	GO:0009611~ response to wounding	8	1.56	0.00107	FGF7, HHEX, DST, SLC1A2, SLC1A3, VASH1, F2RL2, SULF2	4.999
11	GO:0030324~ lung development	9	1.76	0.00112	LAMA5, CCBE1, FGF7, EPAS1, WNT5A, PDPN, GPC3, HES1, STRA6	4.326
12	GO:0043410~ positive regulation of MAPK cascade	12	2.34	0.00114	IL6, FLT1, KSR1, DOK5, INSR, PRKCE, ADORA1, LPAR1, ROR1, ROR2, TMEM106A, AVPI1	3.280
13	GO:0030335~ positive regulation of cell migration	16	3.13	0.00114	FLT1, WNT5B, CSF1, SEMA3D, SPHK1, CAV1, INSR, SEMA6D, THBS1, PLAU, PDPN, CDH13, HAS2, ITGA6, ROR2, HBEGF	2.651
14	GO:0009952~ anterior/posterior pattern specification	9	1.76	0.00148	HHEX, MSX2, TSHZ1, HES1, HOXB8, HOXD10, HOXC11, HOXB6, HOXA4	4.144
15	GO:0008284~ positive regulation of cell proliferation	25	4.88	0.00211	FLT1, CSF1, BNC1, IRS1, SCX, THBS1, PTHLH, FGF5, FGF7, CCND2, HAS2, HES1, TSLP, SPHK1, INSR, GREM1, IL6, KLF5, BAMBI, TNFSF4, MEIS3P1, IL7R, SHANK2, TSPYL5, HBEGF	1.977
16	GO:0007155~ cell adhesion	25	4.88	0.00220	NLGN1, NTM, ITGB2, RND3, THBS1, PCDH18, ROBO1, EFNB1, ALCAM, CYP1B1, HES1, EMB, MYH10, MAGI1, EGFL7, DST, PRKCE, PCDH7, FAP,	1.970

					FEZ1, ITGA11, ADAM12, CDH11, ITGA6, CD44	
17	GO:0001960~ negative regulation of cytokine-mediated signaling pathway	4	0.78	0.00222	ECM1, CAV1, PALM3, IRAK3	14.580
18	GO:1900138~ negative regulation of phospholipase A2 activity	3	0.59	0.00302	ANXA8L1, ANXA1, ANXA8	32.805
19	GO:0042102~ positive regulation of T cell proliferation	7	1.37	0.00361	EFNB1, IL6, ANXA1, TNFSF4, HES1, CD46, CARD11	4.710
20	**GO:0090263~ positive regulation of canonical Wnt signaling pathway**	9	1.76	0.00372	GPRC5B, HHEX, BAMBI, CAV1, GPC3, NRARP, ROR2, ZBED3, SULF2	3.579
21	GO:0072659~ protein localization to plasma membrane	11	2.15	0.00393	RAB3B, IKBKB, LAMA5, CACNB2, EHD3, TMEM88, KCNIP3, WNK3, LYPD1, MYO5A, TNIK	2.988
22	GO:0051092~ positive regulation of NF-kappaB transcription factor activity	11	2.15	0.00393	GREM1, IKBKB, CAV1, SPHK1, WNT5A, ITGB2, TRIM14, ROR1, IRAK3, CARD11, TRIM22	2.988

23	GO:0010035~ response to inorganic substance	4	0.78	0.00437	NT5E, EEF1A2, ADORA1, ENPP1	11.664
24	GO:0009887~ animal organ morphogenesis	10	1.95	0.00444	GREM1, FGF5, TLE3, GREM2, LAMA5, FGF7, TLE1, NTN4, EVL, MEIS3P1	3.170
25	GO:0010575~ positive regulation of vascular endothelial growth factor production	5	0.98	0.00458	CCBE1, IL6, CYP1B1, PTGS2, SULF2	7.290
26	GO:0043123~ positive regulation of I-kappaB kinase/NF-kappaB signaling	12	2.34	0.00471	IKBKB, ECM1, GPRC5B, PRKCE, PELI1, LPAR1, TRIM14, ROR1, LITAF, CARD11, TRIM22, TMEM106A	2.734
27	GO:0050680~ negative regulation of epithelial cell proliferation	7	1.37	0.00486	CDKN1C, TGFBR3, DUSP10, WNT5A, GPC3, EPPK1, MCC	4.437
28	GO:0043154~ negative regulation of cysteine-type endopeptidase activity involved in apoptotic process	7	1.37	0.00486	RPS6KA1, DHCR24, KLF4, PTGS2, THBS1, CD44, AQP1	4.437

29	GO:0045429~ positive regulation of nitric oxide biosynthetic process	6	1.17	0.00505	INSR, ITGB2, NOS1AP, PTX3, KLF4, PTGS2	5.356
30	GO:0035633~ maintenance of permeability of blood-brain barrier	5	0.98	0.00516	IL6, WNK3, PTGS2, TJP2, MFSD2A	7.055

Supplementary Table S4. Kyoto Encyclopedia of Genes and Genomes (KEGG) pathway analysis using DAVID.

No.	Term	Count	%	p-value	Genes	Fold Enrichment
1	hsa04931: Insulin resistance	11	2.15	6.79E-04	IKBKB, IL6, IRS1, GFPT2, INSR, PRKCE, RPS6KA1, ACACB, PPARGC1B, FOXO1, CREB5	3.731
2	hsa04360: Axon guidance	14	2.73	0.0013	WNT5B, SEMA3D, UNC5B, TRPC1, SEMA6D, WNT5A, NTN4, ROBO1, EFNB1, PARD6B, EFNB3, ABLIM3, RASA1, SLIT3	2.818
3	hsa04668: TNF signaling pathway	10	1.95	0.0033	IKBKB, CXCL6, IL6, CSF1, MMP3, FAS, PTGS2, CREB5, MAPK13, MAP3K5	3.270
4	hsa04010: MAPK signaling pathway	17	3.32	0.0063	DUSP2, FLT1, CSF1, BDNF, CACNA2D1, INSR, MAPK13, FGF5, IKBKB, CACNB2, FGF7, DUSP10, MECOM, RASA1, RPS6KA1, FAS, MAP3K5	2.118
5	hsa04151: PI3K-Akt signaling pathway	19	3.71	0.0078	MAGI1, LAMA5, FLT1, CSF1, IRS1, BDNF, INSR, LPAR1, GNG11, THBS1, FGF5, IKBKB, FGF7, IL6, CCND2, ITGA11, ITGA6, IL7R, CREB5	1.966
6	hsa04015: Rap1 signaling pathway	13	2.54	0.0119	MAGI1, FLT1, CSF1, INSR, ITGB2, LPAR1, THBS1, MAPK13, FGF5, PARD6B, FGF7, EVL, PLCB1	2.268

7	**hsa04310: Wnt signaling pathway**	11	2.15	0.0174	TLE3, VANGL2, TLE1, CCND2, WNT5B, BAMBI, WNT5A, ROR1, ROR2, GPC4, PLCB1	2.370
8	hsa05165: Human papillomavirus infection	17	3.32	0.0181	MAGI1, LAMA5, WNT5B, STAT1, MX2, WNT5A, PTGS2, THBS1, FOXO1, IKBKB, PARD6B, CCND2, ITGA11, FAS, ITGA6, HES1, CREB5	1.881
9	hsa04933: AGE-RAGE signaling pathway in diabetic complications	8	1.56	0.0189	IL6, STAT1, PRKCE, AGTR1, PIM1, PLCB1, FOXO1, MAPK13	2.930
10	hsa04068: FoxO signaling pathway	9	1.76	0.0261	IKBKB, IL6, CCND2, HOMER1, IRS1, INSR, IL7R, FOXO1, MAPK13	2.517
11	hsa05200: Pathways in cancer	23	4.49	0.0311	LAMA5, WNT5B, EPAS1, STAT1, WNT5A, LPAR1, PTGS2, GNG11, FOXO1, DLL3, FGF5, IKBKB, FGF7, IL6, CCND2, MECOM, PIM1, AGTR1, FAS, ITGA6, HES1, PLCB1, IL7R	1.587
12	hsa04930: Type II diabetes mellitus	5	0.98	0.0356	IKBKB, IRS1, INSR, PRKCE, HK2	3.981
13	hsa00650: Butanoate metabolism	4	0.78	0.0359	ALDH5A1, BDH1, ABAT, ACADS	5.427
14	hsa04924: Renin secretion	6	1.17	0.0391	PDE3A, ADORA1, AGTR1, PLCB1, CTSB, AQP1	3.185

15	hsa04722: Neurotrophin signaling pathway	8	1.56	0.0431	IKBKB, SHC3, IRS1, BDNF, RPS6KA1, IRAK3, MAPK13, MAP3K5	2.462
16	hsa04152: AMPK signaling pathway	8	1.56	0.0465	PFKFB2, LIPE, IRS1, INSR, ACACB, FOXO1, PFKP, CREB5	2.422

Supplementary Table S5. Gene sets enriched in phenotype U-MSCs (Top 50).

No.	Name	Size	ES	NES	p-val	FDR q-val
1	**GOBP DNA unwinding involved in DNA replication**	21	0.651	2.218	0.000	0.031
2	GOBP ribosome biogenesis	182	0.406	2.171	0.000	0.039
3	GOBP ribonucleoprotein complex biogenesis	275	0.372	2.116	0.000	0.058
4	GOBP cardiac conduction system development	22	0.612	2.086	0.002	0.064
5	GOBP estrogen metabolic process	15	0.685	2.076	0.000	0.057
6	**GOBP cell cycle DNA replication**	33	0.517	2.010	0.000	0.103
7	GOBP positive regulation of BMP signaling pathway	24	0.568	2.000	0.000	0.099
8	**GOBP regulation of DNA templated DNA replication**	39	0.498	1.998	0.000	0.088
9	GOBP protein DNA complex disassembly	17	0.621	1.989	0.002	0.088
10	GOBP chromosome organization	353	0.339	1.988	0.000	0.080
11	**GOBP DNA templated DNA replication**	113	0.397	1.986	0.000	0.074
12	GOBP positive regulation of smoothened signaling pathway	20	0.605	1.972	0.000	0.080
13	GOBP protein localization to chromosome centromeric region	25	0.543	1.968	0.000	0.079
14	GOBP rRNA metabolic process	166	0.375	1.967	0.000	0.073
15	GOBP positive regulation of DNA biosynthetic process	45	0.473	1.953	0.000	0.079
16	GOBP ribosomal large subunit biogenesis	39	0.494	1.942	0.000	0.085
17	GOBP DNA strand elongation	25	0.545	1.914	0.005	0.107
18	GOBP positive regulation of DNA metabolic process	191	0.351	1.907	0.000	0.109
19	GOBP oligodendrocyte differentiation	52	0.440	1.885	0.000	0.129
20	GOBP interstrand cross link repair	24	0.540	1.882	0.006	0.127
21	GOBP regulation of myelination	28	0.521	1.877	0.002	0.127
22	**GOBP DNA replication**	189	0.347	1.875	0.000	0.123
23	GOBP neuropeptide signaling pathway	49	0.447	1.873	0.002	0.120
24	GOBP regulation of establishment of planar polarity	33	0.480	1.854	0.005	0.141
25	GOBP ribonucleoside monophosphate biosynthetic process	17	0.581	1.835	0.009	0.162
26	GOBP positive regulation of transcription by RNA polymerase I	16	0.587	1.832	0.002	0.160
27	GOBP DNA methylation or demethylation	61	0.415	1.817	0.000	0.179
28	GOBP genitalia development	20	0.558	1.815	0.007	0.175
29	GOBP maturation of LSU rRNA	15	0.584	1.800	0.009	0.194
30	GOBP ncRNA processing	251	0.319	1.799	0.000	0.191
31	GOBP DNA dealkylation	25	0.507	1.798	0.004	0.185
32	GOBP oligodendrocyte development	24	0.513	1.797	0.012	0.182

33	GOBP sister chromatid cohesion	31	0.485	1.795	0.000	0.179
34	GOBP maturation of 5.8S rRNA	21	0.536	1.791	0.011	0.180
35	**GOBP cell signaling by Wnt**	257	0.319	1.786	0.000	0.184
36	GOBP chromosome segregation	202	0.328	1.783	0.000	0.183
37	GOBP vascular transport	48	0.429	1.782	0.003	0.180
38	GOBP ligand gated ion channel signaling pathway	15	0.575	1.777	0.010	0.183
39	GOBP negative regulation of lipid catabolic process	15	0.579	1.777	0.011	0.179
40	GOBP establishment of tissue polarity	41	0.433	1.775	0.009	0.177
41	GOBP regulation of mast cell activation involved in immune response	17	0.541	1.775	0.011	0.173
42	GOBP cell surface receptor signaling pathway involved in cell signaling	327	0.306	1.773	0.000	0.171
43	**GOBP non canonical Wnt signaling pathway**	43	0.442	1.773	0.000	0.167
44	GOBP protein localization to chromosome	71	0.392	1.773	0.002	0.164
45	**GOBP regulation of DNA replication**	86	0.374	1.771	0.002	0.164
46	GOBP positive regulation of potassium ion transport	28	0.494	1.759	0.007	0.177
47	GOBP chromosome organization involved in meiotic cell cycle	37	0.454	1.757	0.004	0.176
48	GOBP telomerase RNA localization	16	0.568	1.753	0.024	0.179
49	GOBP mitotic nuclear division	176	0.326	1.751	0.000	0.178
50	GOBP positive regulation of transmembrane receptor protein serine threonine kinase signaling pathway	73	0.381	1.747	0.002	0.180

ES, enrichment score; NES, normalized enrichment score; FDR, false discovery rate.

Supplementary Table S6. Gene sets enriched in phenotype rS-MSCs (Top 50).

No.	Name	Size	ES	NES	p-val	FDR q-val
1	GOBP cortical cytoskeleton organization	38	-0.548	-2.294	0.000	0.026
2	GOBP peroxisome organization	21	-0.657	-2.269	0.000	0.019
3	GOBP lens development in camera type eye	44	-0.514	-2.244	0.000	0.015
4	GOBP cortical actin cytoskeleton organization	25	-0.594	-2.158	0.000	0.044
5	GOBP organelle membrane fusion	76	-0.433	-2.118	0.000	0.058
6	GOBP positive regulation of endothelial cell migration	60	-0.443	-2.086	0.000	0.070
7	GOBP regulation of protein autophosphorylation	23	-0.581	-2.069	0.000	0.071
8	GOBP positive regulation of blood vessel endothelial cell migration	30	-0.531	-2.068	0.000	0.062
9	GOBP membrane fusion	92	-0.395	-2.043	0.000	0.072
10	GOBP positive regulation of peptidyl serine phosphorylation	58	-0.439	-2.032	0.000	0.074
11	GOBP type 2 immune response	19	-0.610	-2.024	0.000	0.073
12	GOBP peroxisomal transport	15	-0.636	-2.021	0.000	0.069
13	GOBP antigen processing and presentation of peptide antigen via MHC class I	17	-0.611	-2.013	0.000	0.070
14	GOBP vacuole organization	119	-0.377	-2.008	0.000	0.067
15	GOBP positive regulation of epithelial cell migration	86	-0.404	-1.994	0.000	0.073
16	GOBP phospholipase C activating G protein coupled receptor signaling pathway	48	-0.444	-1.980	0.000	0.082
17	GOBP positive regulation of protein maturation	16	-0.596	-1.974	0.000	0.081
18	GOBP vesicle docking	45	-0.451	-1.965	0.002	0.084
19	GOBP positive regulation of extrinsic apoptotic signaling pathway	28	-0.511	-1.957	0.002	0.086
20	GOBP lens fiber cell differentiation	21	-0.548	-1.952	0.006	0.085
21	**GOBP positive regulation of autophagy**	86	-0.390	-1.945	0.000	0.088
22	GOBP negative regulation of biomineralization	17	-0.584	-1.939	0.002	0.090
23	GOBP positive regulation of acute inflammatory response	15	-0.598	-1.936	0.002	0.088
24	GOBP positive regulation of vacuole organization	15	-0.623	-1.933	0.002	0.087
25	GOBP motor neuron axon guidance	16	-0.599	-1.922	0.002	0.092
26	GOBP regulation of tube size	78	-0.392	-1.916	0.000	0.093
27	GOBP cellular modified amino acid biosynthetic process	25	-0.515	-1.913	0.009	0.092
28	GOBP vasoconstriction	40	-0.454	-1.907	0.005	0.094
29	**GOBP positive regulation of macroautophagy**	45	-0.442	-1.903	0.002	0.094

30	GOBP positive regulation of animal organ morphogenesis	16	-0.587	-1.901	0.009	0.093
31	GOBP response to angiotensin	19	-0.559	-1.885	0.002	0.105
32	GOBP response to peptide	283	-0.304	-1.883	0.000	0.103
33	GOBP response to prostaglandin	15	-0.591	-1.873	0.004	0.110
34	GOBP secondary metabolic process	33	-0.476	-1.872	0.000	0.107
35	GOBP thyroid hormone metabolic process	19	-0.543	-1.872	0.002	0.104
36	**GOBP macroautophagy**	183	-0.320	-1.869	0.000	0.103
37	GOBP tissue remodeling	95	-0.369	-1.865	0.000	0.105
38	GOBP regulation of protein maturation	37	-0.456	-1.860	0.000	0.106
39	GOBP cranial nerve morphogenesis	19	-0.541	-1.857	0.009	0.107
40	GOBP positive regulation of inflammatory response	77	-0.374	-1.856	0.000	0.105
41	GOBP vesicle localization	118	-0.351	-1.853	0.003	0.105
42	GOBP membrane docking	61	-0.397	-1.836	0.000	0.119
43	GOBP response to amyloid beta	31	-0.473	-1.835	0.000	0.117
44	GOBP exocytic process	51	-0.408	-1.833	0.000	0.117
45	GOBP negative regulation of ossification	17	-0.547	-1.831	0.007	0.116
46	GOBP positive regulation of protein localization to cell periphery	42	-0.435	-1.829	0.007	0.115
47	GOBP foam cell differentiation	21	-0.519	-1.821	0.008	0.120
48	GOBP regulation of PH	52	-0.407	-1.816	0.002	0.123
49	GOBP regulation of bone mineralization	54	-0.393	-1.814	0.000	0.124
50	GOBP positive regulation of morphogenesis of an epithelium	19	-0.528	-1.809	0.002	0.127
